# Supplementary material for: Quality of medicines for Cardio-Vascular Diseases (CVDs) in the Ethiopian border with Kenya: The case of enalapril maleate and furosemide tablet quality in Borena and Gedeo zones
Source: PLOS Glob Public Health. 2024 Jul 15;4(7):e0003104. doi: 10.1371/journal.pgph.0003104 (PMC11249254; doi:10.1371/journal.pgph.0003104)
Supplement: S1 Fig — (DOC) [file pgph.0003104.s001.doc]

**S1Fig: Visual Observation defects:** Failed visual inspection report of enalapril maleate and furosemide tablets

| Generic name (Brand) | Batch number | Mfg. Date  Exp. Date | Place of collection | Physical characteristics of the products | Images of failed visual inspection samples |
| --- | --- | --- | --- | --- | --- |
| Frusomide  **(Leprusid)** | 78576 | 03/21  02/24 | Moyale | Black spots on the tablet | 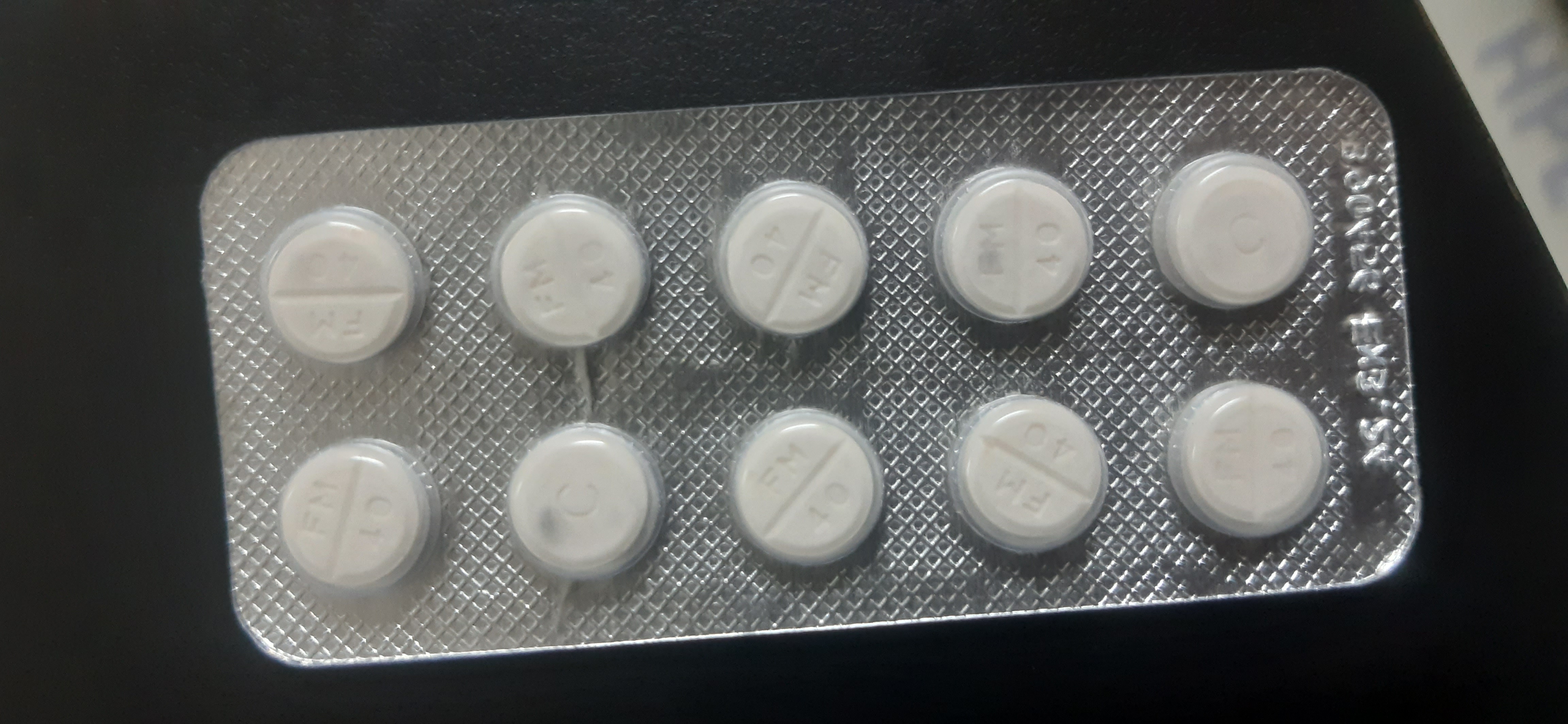 |
| Enalapril  (Korandil) | 90786 | 11/2020  11/2023 | Yabelo | -Very hard to open from the blister (stick with the blister)  - Tablet shattering during opening | 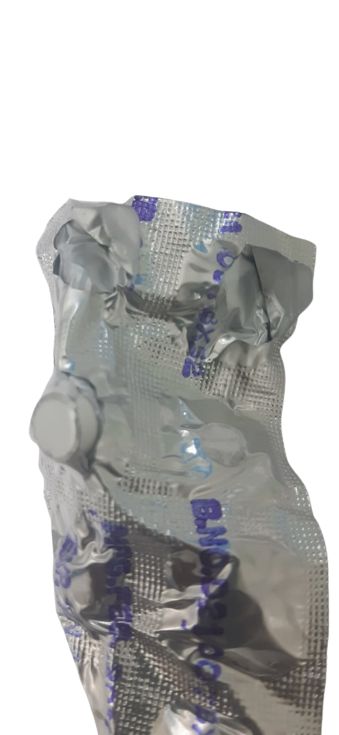 |
| Enalapril  (Enali-SSP) | 06120110010 | 11/2020  10/2022 | Yabelo | **-**Two tablets (one slightly cracked) tablets  exist together.  **-**Powders around the blisters | 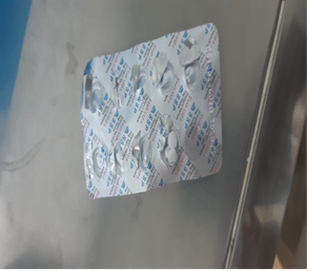 |
| Furosemide  (Fusix) | 1060373 | 06/21  06/24 | Yabelo | **-**Breaks & splits on hand during opening of the blister. | 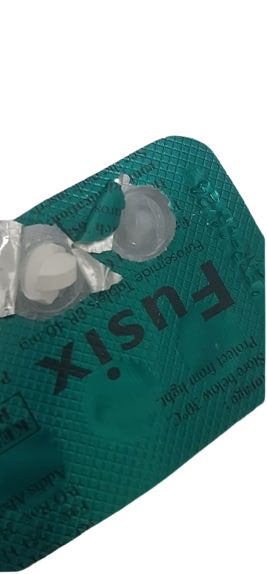 |
| Furosemide  (Fruz) | BPL763 | 04/2021  03/2024 | Yirgachefe | **-**Breaks, & cracks around the edge.  -Powdered inside the blister | 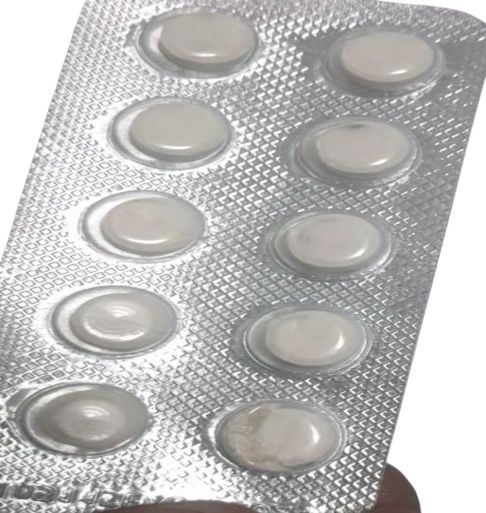 |
| Frusemide | 2105115 | 05/2021  04/2024 | Moyale | -Breaks & splits inside and on hand during opening of the blister. | 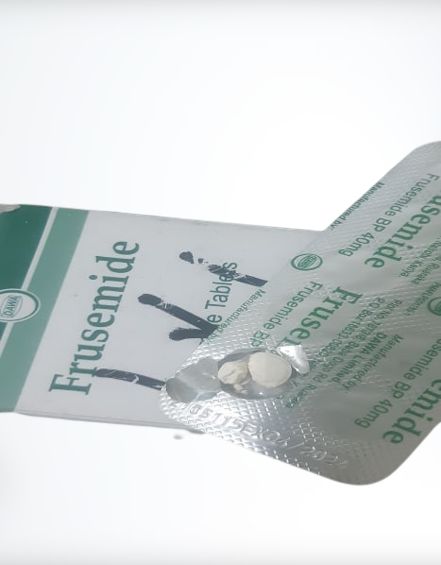 |
| Furosemide  (Fruz) | BPL763 | 04/2021  03/2024 | Dilla | Two tablets overlap in one primary packaging. | 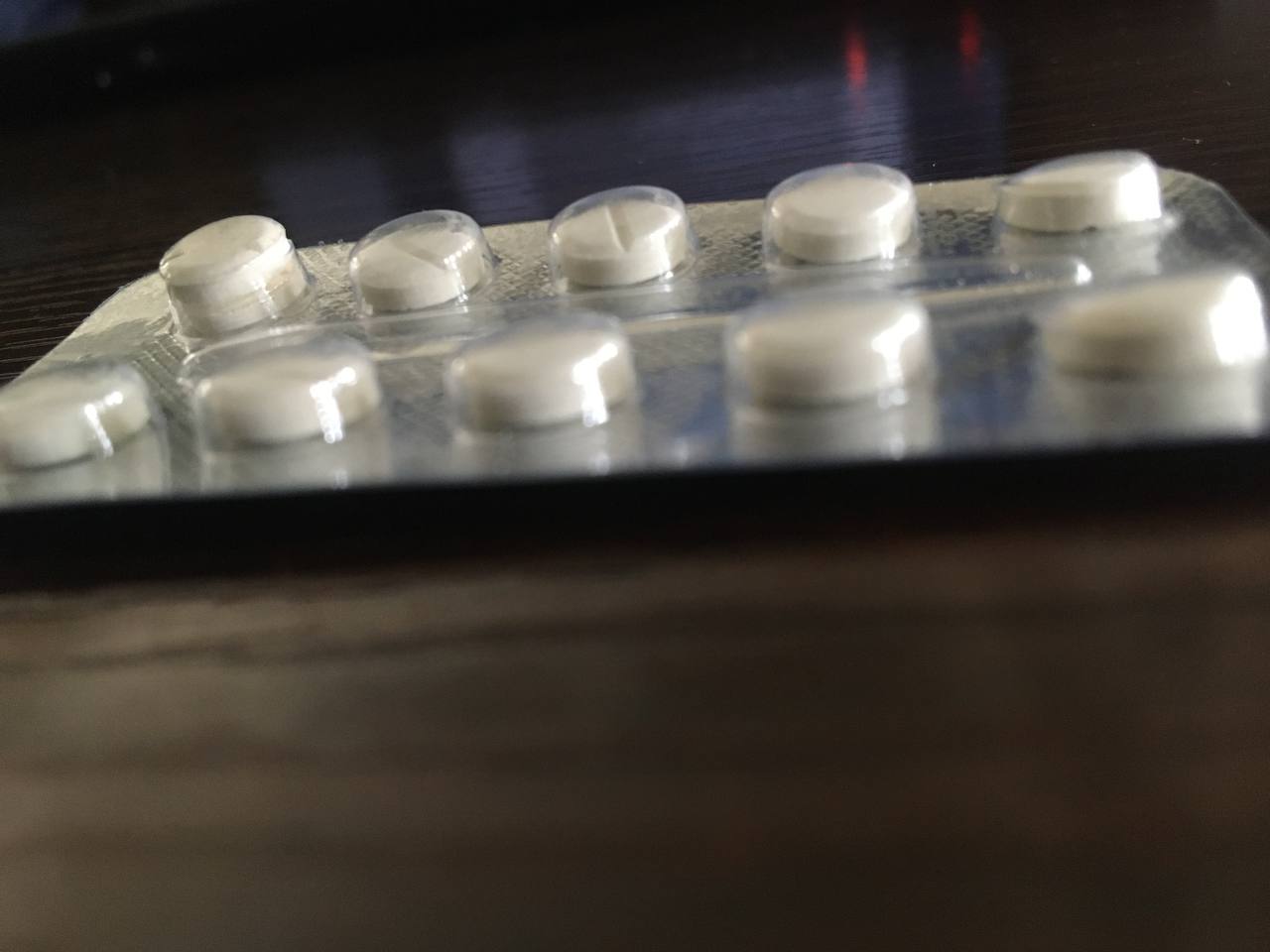 |
