# Supplementary figures and images for: Quality of medicines for Cardio-Vascular Diseases (CVDs) in the Ethiopian border with Kenya: The case of enalapril maleate and furosemide tablet quality in Borena and Gedeo zones
Source: PLOS Glob Public Health. 2024 Jul 15;4(7):e0003104. doi: 10.1371/journal.pgph.0003104 (PMC11249254; doi:10.1371/journal.pgph.0003104)

S2Fig. Sample conducting the test in the laminar flow hood


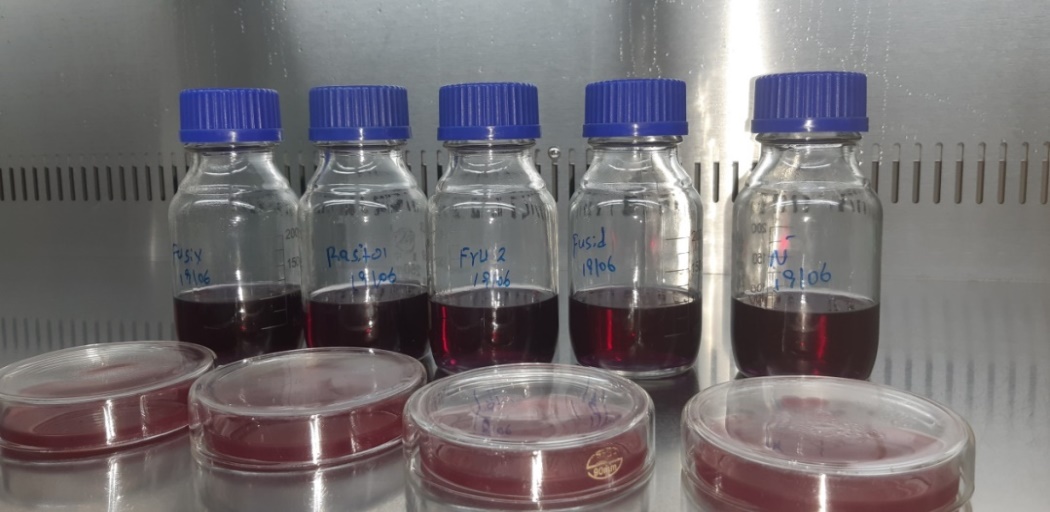

Supplement: S2 Fig — (DOC) [file pgph.0003104.s002.doc]
