# Supplementary material for: Quality of medicines for Cardio-Vascular Diseases (CVDs) in the Ethiopian border with Kenya: The case of enalapril maleate and furosemide tablet quality in Borena and Gedeo zones
Source: PLOS Glob Public Health. 2024 Jul 15;4(7):e0003104. doi: 10.1371/journal.pgph.0003104 (PMC11249254; doi:10.1371/journal.pgph.0003104)
Supplement: S3 Fig — (DOC) [file pgph.0003104.s003.doc]

S3Fig. Results of TAMC, TYMC and streaking plate method (Photo)


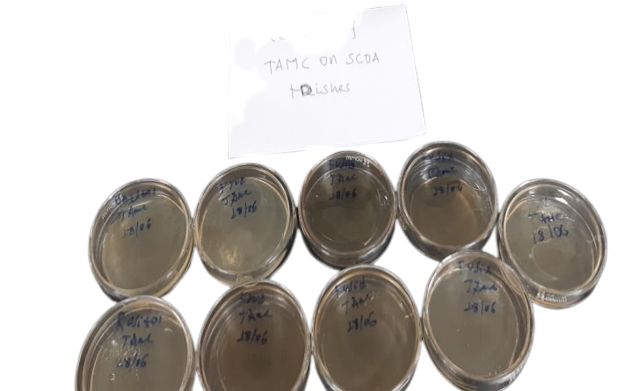

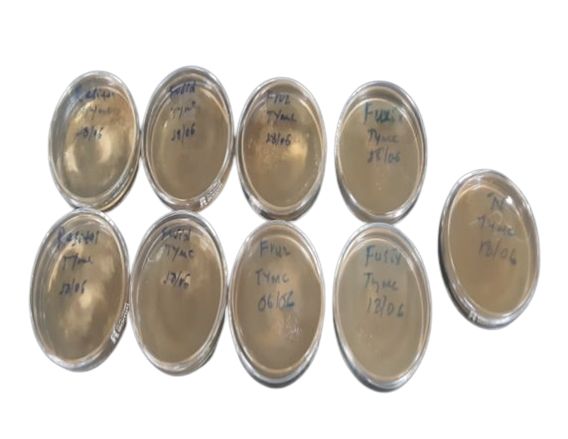

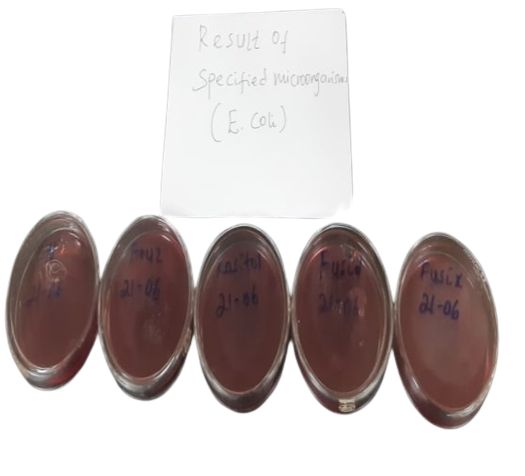

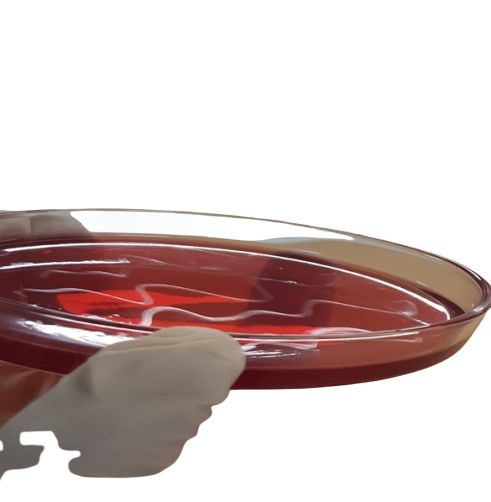


Result of TYMC on SDA dishes

Result of TAMC on SDA dishes

Result of *E.coli*

Streak plate method
