# Supplementary material for: Quality of medicines for Cardio-Vascular Diseases (CVDs) in the Ethiopian border with Kenya: The case of enalapril maleate and furosemide tablet quality in Borena and Gedeo zones
Source: PLOS Glob Public Health. 2024 Jul 15;4(7):e0003104. doi: 10.1371/journal.pgph.0003104 (PMC11249254; doi:10.1371/journal.pgph.0003104)
Supplement: S1 File — (DOC) [file pgph.0003104.s004.doc]

S1 File. Detail information on enalapril maleate samples used for the study.

| S.No. | Code and brand | Method of analysis | Dosage/Strength | Lot/batch No. | Town purchased | Mfg. Date | Ex. Date | Manufacturer company | Country of origin |
| --- | --- | --- | --- | --- | --- | --- | --- | --- | --- |
| 01 | EY-01  (Enali-SSP) | USP | 5mg | 06120110010 | Yabelo | 11/20 | 10/22 | Shansheng pharamaceutical Plc | Ethiopia |
| 02 | EY-04  (Enali-SSP) | USP | 5mg | 06121060010 | Yabelo | 06/21 | 05/23 | Shansheng pharamaceutical Plc | Ethiopia |
| 03 | EY-05 (Korandil) | USP | 5mg | 90786 | Yabelo | 11/20 | 11/23 | Remedica Limited | Cyprus |
| 04 | EY-06 (Enali-SSP) | USP | 5mg | 06121070020 | Yabelo | 07/21 | 06/23 | Shansheng pharamaceutical Plc | Ethiopia |
| 05 | ED-11 (Enaril) | USP | 5mg | SDJ662 | Dilla | 02/21 | 01/23 | Beximco Pharmaceuticals Limited | Bangladesh |
| 06 | EM-04 (Acepril) | USP | 5mg | 75034 | Moyale | 07/19 | 06/22 | Laboratory and Allied Limited | Kenya |
| 07 | EM-08  (Encardil) | USP | 5mg | D10011 | Moyale | 01/21 | 12/23 | Medley Pharmaceuticals Limited | India |
| 08 | EM-08’1  (Encardil) | USP | 5mg | D00924 | Moyale | 11/23 | 10/23 | Medley Pharmaceuticals Limited | India |
| 09 | EM-09  (Encardil) | USP | 5mg | D10122 | Moyale | 01/21 | 12/23 | Medley Pharmaceuticals Limited | India |
| 10 | EM-10’1 (Enaril) | USP | 5mg | SDJ662 | Moyale | 10/20 | 10/22 | Beximco Pharmaceuticals Limited | Bangladesh |
| 11 | EM-10  (Enaril) | USP | 5mg | SEB243 | Moyale | 02/21 | 01/23 | Beximco Pharmaceuticals Limited | Bangladesh |
| 12 | EMG-01 (Envas) | USP | 5mg | D21012BX52 | Moyale Primary  Hospital | 03/21 | 02/23 | Cadila Pharmaceuticals Plc | Ethiopia |
| 13 | EY-06 (Enali-SSP) | USP | 5mg | 06120110010 | Yabelo General  Hospital | 11/20 | 10/22 | Sansheng Pharmaceutical Plc | Ethiopia |
| 14 | EYC-01’1  (Envas) | USP | 5mg | D21007BX52 | Yirgachefe | 02/21 | 01/23 | Cadila Pharmaceuticals Plc | Ethiopia |
| 15 | EYCG-01 (Envas) | USP | 5mg | D21031BX52 | Yirgachefe Primary Hospital | 08/21 | 07/23 | Cadila Pharmaceuticals Plc | Ethiopia |
| 16 | EDG-01 (Enali-SSP) | USP | 5mg | 06121060010 | Dilla General  Hospital | 06/21 | 05/23 | Sansheng Pharmaceutical Plc | Ethiopia |
| 17 | ED-08 (Envas) | USP | 5mg | D21024BX52 | Dilla | 06/21 | 06/23 | Cadila Pharmaceuticals Plc | Ethiopia |
| 18 | EYC-01 (Enali-SSP) | USP | 5mg | 0612109003 | Yirgachefe | 09/21 | 08/23 | Sansheng Pharmaceutical Plc | Ethiopia |
| 19 | EYC-01’1  (Envas) | USP | 5mg | D21007BX52 | Yirgachefe | 02/21 | 01/23 | Cadila Pharmaceuticals Plc | Ethiopia |
| 20 | ED-02 (Envas) | USP | 5mg | D21007BX52 | Dilla | 08/21 | 07/23 | Cadila Pharmaceuticals Plc | Ethiopia |
| 21 | EW-01 (Envas) | USP | 5mg | D21007BX52 | Wenago | 02/21 | 01/23 | Cadila Pharmaceuticals Plc | Ethiopia |
| 22 | ED-04 (Enali-SSP) | USP | 5mg | 06121070020 | Dilla | 07/21 | 06/23 | Sansheng Pharmaceutical Plc | Ethiopia |
| 23 | EG-02 (Enali-SSP) | USP | 5mg | 06121060030 | Gedeb | 12/21 | 05/23 | Sansheng Pharmaceutical Plc | Ethiopia |
| 24 | EGG-01 (Enali-SSP) | USP | 5mg | 06121070030 | Gedeb Primary  Hospital | 07/21 | 06/23 | Sansheng Pharmaceutical Plc | Ethiopia |
| 25 | EM-03 | USP | 5mg | D21024BX52 | Moyale | 07/21 | 06/23 | Cadila Pharmaceuticals Plc | Ethiopia |
