# Supplementary material for: Quality of medicines for Cardio-Vascular Diseases (CVDs) in the Ethiopian border with Kenya: The case of enalapril maleate and furosemide tablet quality in Borena and Gedeo zones
Source: PLOS Glob Public Health. 2024 Jul 15;4(7):e0003104. doi: 10.1371/journal.pgph.0003104 (PMC11249254; doi:10.1371/journal.pgph.0003104)
Supplement: S2 File — (DOC) [file pgph.0003104.s005.doc]

S2 File. Detail information on furosemide samples used for the study.

| 1 | FY-02  (Fusix) | BP | 40mg | 1060373 | Yabelo | 06/21 | 06/24 | Ethiopian Pharmaceutical Manufacturing(EPHARM) | Ethiopia |
| --- | --- | --- | --- | --- | --- | --- | --- | --- | --- |
| 2 | FY-03  (Fusix) | BP | 40mg | 1060423 | Yabelo | 06/21 | 06/24 | Ethiopian Pharmaceutical Manufacturing(EPHARM) | Ethiopia |
| 3 | FM-01 (Fusix) | BP | 40mg | 1060493 | Moyale | 06/21 | 06/24 | Ethiopian Pharmaceutical Manufacturing | Ethiopia |
| 4 | FM-02  (Frusemide) | BP | 40mg | 2105115 | Moyale | 05/21 | 04/24 | DAWA Limited | Kenya |
| 5 | FM-05  (Frusemide) | BP | 40mg | 71225 | Moyale | 11/17 | 10/22 | Cosmos Limited | Kenya |
| 6 | FM-05’1  (Furosemide) | BP | 40mg | 90456 | Moyale | 04/19 | 03/24 | Cosmos Limited | Kenya |
| 7 | FM-06  (Furosemide) | USP | 40mg | 210110 | Moyale | 01/21 | 12/25 | Cosmos Limited | Kenya |
| 8 | FM-07  (Fruz) | BP | 40mg | BPL818 | Moyale | 06/21 | 05/24 | Biopharma Limited | Kenya |
| 9 | FM-10 (Lefrusid) | USP | 40mg | 78576 | Moyale | 03/21 | 02/24 | Laboratory and Allied Limited | Kenya |
| 10 | FMG-01  (Fusid) | USP | 40mg | 31605 | Moyale Primary  Hospital | 04/20 | 04/22 | Addis Pha rmaceuticals Factory S.C | Ethiopia |
| 11 | FYG-01  (Fusix) | BP | 40mg | 1070103 | Yabelo General  Hospital | 07/21 | 07/23 | Ethiopian Pha rmaceutical Manufacturing | Ethiopia |
| 12 | FD-01  (Rasitol) | USP | 40mg | FCOO2 | Dilla | 03/21 | 03/24 | Y.S.P. Industries (M) | Malaysia |
| 13 | FD-03  (Fusix) | BP | 40mg | 1070023 | Dilla | 07/21 | 07/24 | Ethiopian Pha rmaceutical Manufacturing | Ethiopia |
| 14 | FD-04  (Furo-Denk) | USP | 40mg | 9ZP | Dilla | 05/21 | 05/24 | Denk Pharma GmbH & co.KG | Germany |
| 15 | FD-10 (Fusix) | BP | 40mg | 1060513 | Dilla | 05/21 | 06/24 | Ethiopian Pha rmaceutical Manufacturing | Ethiopia |
| 16 | FD-04 (Fusix) | BP | 40mg | 1060423 | Dilla | 06/21 | 06/24 | Ethiopian Pha rmaceutical Manufacturing | Ethiopia |
| 17 | FD-09’2  (Fruz) | BP | 40mg | BPL763 | Dilla | 04/21 | 03/24 | Biopharma Limited | Kenya |
| 18 | FDG-02  (Novartis Access-Furosemide) | USP | 40mg | LJ5560 | Dilla General  Hospital | 01/21 | 12/22 | Sandoz Private Limited MIDC, India | Germany |
| 19 | FDG-01’1  (Novartis Access-Furosemide) | USP | 40mg | LJ559 | Dilla General  Hospital | 01/21 | 12/22 | Sandoz Private Limited MIDC, India | Germany |
| 20 | FDG-01  (Novartis Access-Furosemide) | USP | 40mg | LJ5561 | Dilla General  Hospital | 01/21 | 12/22 | Sandoz Private Limited MIDC, India | Germany |
| 21 | FD-10’1  (Fusix) | BP | 40mg | 1060413 | Dilla | 06/21 | 06/24 | Ethiopian Pha rmaceutical Manufacturing | Ethiopia |
| 22 | FYC-01  (Rasitol) | USP | 40mg | FCOO2 | Yirgachefe | 03/21 | 03/24 | Y.S.P.Industries (M) SDN | Malaysia |
| 23 | FYC-02  (Fruz) | BP | 40mg | BPL763 | Yirgachefe | 04/21 | 03/24 | Biopharma Limited | Ethiopia |
| 24 | FYCG-01  (Fusix) | BP | 40mg | 1070013 | Yirgachefe Primary Hospital | 07/21 | 07/24 | Ethiopian Pha rmaceutical Manufacturing | Ethiopia |
| 25 | FW-01 (Fruz) | BP | 40mg | BPL763 | Wenago | 04/21 | 03/24 | Biopharma Limited | Kenya |
| 26 | FD-11 (Fusix) | BP | 40mg | 1070203 | Dilla | 07/21 | 07/23 | Ethiopian Pha rmaceutical Manufacturing | Ethiopia |
| 27 | FG-02  (Rasitol) | USP | 40mg | FGOO4 | Gedeb | 07/21 | 07/24 | Y.S.P.Industries (M) SDN | Malaysia |
| 28 | FGG-01 (Fusix) | BP | 40mg | 1070063 | Gedeb Primary  Hospital | 07/21 | 07/24 | Ethiopian Pha rmaceutical Manufacturing | Ethiopia |
| 29 | FG-01  (Fusix) | BP | 40mg | 1060353 | Gedeb | 06/21 | 06/24 | Ethiopian Pha rmaceutical Manufacturing | Ethiopia |
| 30 | FG-03  (Fusix) | BP | 40mg | 1060453 | Gedeb | 06/21 | 06/24 | Ethiopian Pha rmaceutical Manufacturing | Ethiopia |
