# Supplementary material for: Quality of medicines for Cardio-Vascular Diseases (CVDs) in the Ethiopian border with Kenya: The case of enalapril maleate and furosemide tablet quality in Borena and Gedeo zones
Source: PLOS Glob Public Health. 2024 Jul 15;4(7):e0003104. doi: 10.1371/journal.pgph.0003104 (PMC11249254; doi:10.1371/journal.pgph.0003104)
Supplement: S3 File — (DOC) [file pgph.0003104.s006.doc]

S3 File. General information on number, area and source of collected sample

| **Source of collected sample** | **Gedeo zone** | | | **Borena zone** | | |
| --- | --- | --- | --- | --- | --- | --- |
|  | **Dilla** | **Wenago** | **Yirgachefe** | **Gedeb** | **Yabelo** | **Moyale** |
| Drug store | 9 | 2 | 5 | 4 | 6 | 12 |
| Pharmacy | 3 | **--** | **--** | -- | -- | 2 |
| Hospital pharmacy | 4*** | **--** | 2* | 2* | 2** | 2* |
| Total samples | 16 | 2 | 7 | 6 | 8 | 16 |

* = Primary Hospital, ** = General Hospital, ***=Referral Hospital, ‘^----"^= No samples collected
