# Supplementary material for: Quality of medicines for Cardio-Vascular Diseases (CVDs) in the Ethiopian border with Kenya: The case of enalapril maleate and furosemide tablet quality in Borena and Gedeo zones
Source: PLOS Glob Public Health. 2024 Jul 15;4(7):e0003104. doi: 10.1371/journal.pgph.0003104 (PMC11249254; doi:10.1371/journal.pgph.0003104)
Supplement: S4 File — (DOC) [file pgph.0003104.s007.doc]

S4 File. General information on samples used for the study

| **S. No.** | **Brand name** | **Country of manufacturer** | **Manufacturer** |
| --- | --- | --- | --- |
| 1 | Rasitol***** | Malaysia | Y.S.P. Industries (M) SDN.BHD. |
| 2 | Fusix***** | Ethiopia | EPHARM |
| 3 | Furosemide | Mumbai, India | Sandoz Private Limited MIDC |
| 4 | Fruz***** | Kenya | Biopharma Limited |
| 5 | Lefrusid***** | Kenya | Laboratory & Allied |
| 6 | Fusid***** | Ethiopia | Addis Pharmaceuticals |
| 7 | Furo-denk | Germany | Denk Pharma GmbH & Co.KG |
| 8 | Enali-SSP | Ethiopia | Sansheng Pharmaceutical |
| 9 | Korandil-5 | Cyprus | Remedica |
| 10 | Acepril***** | Kenya | Laboratory & Allied |
| 11 | Encardil***** | Mumbai, India | Medley Pharmaceuticals |
| 12 | Enaril***** | Bangladesh | Beximco Pharmaceuticals |
| 13 | Envas-5 | Ethiopia | Cadila Pharmaceuticals |

***** Unregistered drugs by EFDA
