# Supplementary material for: Quality of medicines for Cardio-Vascular Diseases (CVDs) in the Ethiopian border with Kenya: The case of enalapril maleate and furosemide tablet quality in Borena and Gedeo zones
Source: PLOS Glob Public Health. 2024 Jul 15;4(7):e0003104. doi: 10.1371/journal.pgph.0003104 (PMC11249254; doi:10.1371/journal.pgph.0003104)
Supplement: S5 File — (DOC) [file pgph.0003104.s008.doc]

S5 File. Identification test results of furosemide tablets (USP-2020)

| **S.No** | **Sample code** | **Absorbance maxima** | | **Absorbance minima** | |
| --- | --- | --- | --- | --- | --- |
|  |  | **Wavelength (nm)** | **Absorbance** | **Wavelength (nm)** | **Absorbance** |
| 1 | Furosemide standard | 227.00 | 0.798 | 332.420 | 0.071 |
| 2 | FDG-01’2 | 227.102 | 0.781 | 332.783 | 0.069 |
| 3 | FG-02 | 227.119 | 0.791 | 332.986 | 0.068 |
| 4 | FM-10 | 227.112 | 0.733 | 332.33 | 0.060 |
| 5 | FYC-02 | 226.85 | 0.737 | 332.38 | 0.058 |
| 6 | FD-09’2 | 226.82 | 0.746 | 332.23 | 0.059 |
| 7 | FM-01 | 226.80 | 0.778 | 332.99 | 0.063 |
| 8 | FD-01 | 226.904 | 0.890 | 331.769 | 0.155 |
| 9 | FDG-01 | 226.987 | 0.903 | 331.277 | 0.152 |
| 10 | FDG-02 | 226.580 | 1.157 | 327.986 | 0.364 |
| 11 | FYC-01 | 226.529 | 0.926 | 331.232 | 0.056 |
| 12 | FD-04 | 226.479 | 0.908 | 331.239 | 0.150 |
| 13 | FMG-01 | 226.575 | 0.915 | 331.261 | 0.152 |
| 14 | FY-03 | 224.935 | 0.879 | 331.143 | 0.151 |
| 15 | FD-10 | 225.551 | 0.884 | 331.725 | 0.149 |
| 16 | FGG-01 | 224.69 | 0.879 | 328.107 | 0.241 |
| 17 | FM-02 | 225.651 | 0.872 | 331.096 | 0.101 |
| 18 | FM-05’1 | 225.013 | 0.879 | 331.440 | 0.141 |
| 19 | FW-01 | 226.565 | 0.803 | 332.298 | 0.098 |
| 20 | FM-07 | 225.128 | 0.876 | 327.904 | 0.151 |
