# Supplementary material for: Quality of medicines for Cardio-Vascular Diseases (CVDs) in the Ethiopian border with Kenya: The case of enalapril maleate and furosemide tablet quality in Borena and Gedeo zones
Source: PLOS Glob Public Health. 2024 Jul 15;4(7):e0003104. doi: 10.1371/journal.pgph.0003104 (PMC11249254; doi:10.1371/journal.pgph.0003104)
Supplement: S6 File — (DOC) [file pgph.0003104.s009.doc]

S6 File. Identification test results of furosemide tablets (BP-2020)

| **S.No** | **Sample code** | **Absorbance maxima** | | **Absorbance minima** | |
| --- | --- | --- | --- | --- | --- |
|  |  | **Wavelength (nm)** | **Absorbance** | **Wavelength (nm)** | **Absorbance** |
|  | Furosemide standard | 227.255 | 0.833 | 269.788 | 0.445 |
| 1 | FD-10’1 | 227.259 | 0.832 | 269.812 | 0.446 |
| 2 | FD-11 | 227.202 | 0.841 | 269.813 | 0.449 |
| 3 | FG-01 | 227.079 | 0.793 | 269.840 | 0.421 |
| 4 | FYG-01 | 226.935 | 0.890 | 269.798 | 0.443 |
| 5 | FD-03 | 228.565 | 0.793 | 269.612 | 0.447 |
| 6 | FY-02 | 228.524 | 0.802 | 269.813 | 0.445 |
| 7 | FG-03 | 228.418 | 0.764 | 269.810 | 0.423 |
| 8 | FM-06 | 227.188 | 0.851 | 269.565 | 0.461 |
| 9 | FYC-03 | 226.609 | 0.805 | 269.816 | 0.422 |
| 10 | FYCG-01 | 227.029 | 0.817 | 269.804 | 0.433 |
