# Supplementary material for: Quality of medicines for Cardio-Vascular Diseases (CVDs) in the Ethiopian border with Kenya: The case of enalapril maleate and furosemide tablet quality in Borena and Gedeo zones
Source: PLOS Glob Public Health. 2024 Jul 15;4(7):e0003104. doi: 10.1371/journal.pgph.0003104 (PMC11249254; doi:10.1371/journal.pgph.0003104)
Supplement: S7 File — (DOC) [file pgph.0003104.s010.doc]

S7 File. Assay value, collection site, brands and batches of enalapril maleate

| **S.No** | **Sample code** | **Brand name** | **Batch number** | **Sample site** | **Assay value (%)** | **Conclusion** |
| --- | --- | --- | --- | --- | --- | --- |
| 1 | ED-08 | Envas-5 | D21024BX52 | Dilla | 98.1604 | Passed |
| 2 | EY-06 | Enali-5 | 06121070020 | Yabelo | 97.7459 | Passed |
| 3 | EYC-01 | Enali-5 | 06121090030 | Yirgachefe | 99.3894 | Passed |
| 4 | EM-01 | Enaril | SDJ662 | Moyale | 98.6325 | Passed |
| 5 | EM-03 | Envas-5 | D21024BX52 | Moyale | 99.9922 | Passed |
| 6 | EM-08 | Encardil | D00924 | Moyale | 102.1315 | Passed |
| 7 | EY-04 | Enali-SSP | 06121060010 | Yabelo | 97.1815 | Passed |
| 8 | EYG-01 | Enali-SSP | 06120110010 | Yabelo** | 96.3889 | Passed |
| 9 | EGG-01 | Lefrusid | 78576 | Gedeb* | 97.2538 | Passed |
| 10 | EM-10'1 | Enaril | SDJ662 | Moyale | 98.8543 | Passed |
| 11 | EDG-01 | Enali-SSP | 06121060010 | Dilla*** | 98.2264 | Passed |
| 12 | EY-01 | Enali-SSP | 061201100110 | Yabelo | 98.6423 | Passed |
| 13 | EM-07 | Encardil | D00923 | Moyale | 91.3481 | Passed |
| 14 | EG-02 | Enali-SSP | 06121060030 | Gedeb | 104.5310 | Passed |
| 15 | ED-09 | Envas | D21031BX52 | Dilla | 101.2320 | Passed |
| 16 | ED-11 | Enaril | SDJ662 | Dilla | 98.2530 | Passed |
| 17 | EM-10 | Enaril | SEB243 | Moyale | 100.8180 | Passed |
| 18 | ED-01 | Enali-SSP | 06121070020 | Dilla | 101.9990 | Passed |
| 19 | EMG-01 | Envas | D21012BX52 | Moyale* | 100.6270 | Passed |
| 20 | EM-04 | Acepril | 75034 | Moyale | 103.2055 | Passed |
| 21 | EW-01 | Envas | D21007BX52 | Wenago | 99.9110 | Passed |
| 22 | EYC-01 | Enali-SSP | 06121090030 | Yirgachefe | 102.3805 | Passed |
| 23 | EY-05 | Korandil | 90786 | Yabelo | 98.7445 | Passed |
| 24 | ED-02 | Envas-5 | D21007BX52 | Dilla | 99.9320 | Passed |
| 25 | ED-04 | Enali-SSP | 06122090030 | Dilla | 103.7455 | Passed |

* = Primary Hospital, ** = General Hospital, ***= Referral Hospital
