# Supplementary material for: Quality of medicines for Cardio-Vascular Diseases (CVDs) in the Ethiopian border with Kenya: The case of enalapril maleate and furosemide tablet quality in Borena and Gedeo zones
Source: PLOS Glob Public Health. 2024 Jul 15;4(7):e0003104. doi: 10.1371/journal.pgph.0003104 (PMC11249254; doi:10.1371/journal.pgph.0003104)
Supplement: S8 File — (DOC) [file pgph.0003104.s011.doc]

S8 File. Assay value, brand name and sample site of furosemide tablets (BP 2020)

| **S.No.** | **Brand name** | **Batch No.** | **Sample site** | **Sample code** | **Absorbance at fixed λ (271 nm)** | **Assay**  **value (%)** | **Conclusion** |
| --- | --- | --- | --- | --- | --- | --- | --- |
| 1 | Fusix | 1060423 | Dilla | FD-10’1 | 0.461 | 98.76 | Passed |
| 2 | Fusix | 1070203 | Dilla | FD-11 | 0.472 | 101.11 | Passed |
| 3 | Fusix | 1060353 | Gedeb | FG-01 | 0.446 | 95.54 | Passed |
| 4 | Fusix | 1070103 | Yabelo****** | FYG-01 | 0.469 | 100.47 | Passed |
| 5 | Fusix | 1070023 | Dilla | FD-03 | 0.459 | 98.33 | Passed |
| 6 | Fusix | 1060373 | Yabelo | FY-02 | 0.446 | 95.54 | Passed |
| 7 | Fusix | 1060453 | Gedeb | FG-03 | 0.431 | **92.33** | **Failed** |
| 8 | Furosemide | 210110 | Moyale | FM-06 | 0.472 | 101.11 | Passed |
| 9 | Fusix | 1060413 | Yirgachefe | FYC-03 | 0.433 | **92.76** | **Failed** |
| 10 | Fusix | 1070013 | Yirgachefe* | FYCG-01 | 0.453 | 97.04 | Passed |

*****= Primary Hospital, ****** = General Hospital, λ = Wavelength
