# Supplementary material for: Quality of medicines for Cardio-Vascular Diseases (CVDs) in the Ethiopian border with Kenya: The case of enalapril maleate and furosemide tablet quality in Borena and Gedeo zones
Source: PLOS Glob Public Health. 2024 Jul 15;4(7):e0003104. doi: 10.1371/journal.pgph.0003104 (PMC11249254; doi:10.1371/journal.pgph.0003104)
Supplement: S9 File — (DOC) [file pgph.0003104.s012.doc]

S9 File. Assay value, brand name and sample site of furosemide (USP 43-2020)

| **S.No.** | **Brand name** | **Batch number** | **Sample site** | **Sample code** | **Assay value (%)** | **Conclusion** |
| --- | --- | --- | --- | --- | --- | --- |
| 1 | Rasitol | FC002 | Dilla | FD-01 | 97.4112 | Passed |
| 2 | Furosemide | LJ5561 | Dilla** | FDG-01 | 98.9663 | Passed |
| 3 | Furosemide | LJ5560 | Dilla** | FDG-02 | 99.2893 | Passed |
| 4 | Rasitol | FC002 | Yirgachefe | FYC-01 | 100.3731 | Passed |
| 5 | Furo-Denk | 9ZP | Dilla | FD-04 | 100.2532 | Passed |
| 6 | Fusid | 31605 | Moyale* | FMG-01 | 97.3203 | Passed |
| 7 | Furosemide | LJ5559 | Dilla** | FDG-01’1 | 98.8820 | Passed |
| 8 | Rasitol | FG004 | Gedeb | FG-02 | 100.7973 | Passed |
| 9 | Furosemide | 71225 | Moyale | FM-05 | 100.6570 | Passed |
| 10 | Furosemide | 210110 | Moyale | FM-06 | 96.2317 | Passed |
| 11 | Lefrusid | 78576 | Moyale | FM-10 | 95.1390 | Passed |
| 12 | Fruz | BPL 763 | Dilla | FD-09’2 | 94.8711 | Passed |
| 13 | Fruz | BPL763 | Yirgachefe | FYC-02 | 100.2708 | Passed |
| 14 | Fusix | 1060423 | Yabelo | FY-03 | 96.2323 | Passed |
| 15 | Fusix | 1060513 | Dilla | FD-10 | 100.8208 | Passed |
| 16 | Fusix | 1070063 | Gedeb* | FGG-01 | 96.6823 | Passed |
| 17 | Frusemide | 2105115 | Moyale | FM-02 | 97.0678 | Passed |
| 18 | Furosemide | 90456 | Moyale | FM-05’1 | 100.6555 | Passed |
| 19 | Fruz | BPL763 | Wenago | FW-01 | 95.3575 | Passed |
| 20 | Fruz | BPL818 | Moyale | FM-07 | 91.5245 | Passed |

*= Primary Hospital, **= Referral Hospital
