# Supplementary material for: Quality of medicines for Cardio-Vascular Diseases (CVDs) in the Ethiopian border with Kenya: The case of enalapril maleate and furosemide tablet quality in Borena and Gedeo zones
Source: PLOS Glob Public Health. 2024 Jul 15;4(7):e0003104. doi: 10.1371/journal.pgph.0003104 (PMC11249254; doi:10.1371/journal.pgph.0003104)
Supplement: S10 File — (DOC) [file pgph.0003104.s013.doc]

**S10 File**. Hardness, friability and disintegration test results of enalapril maleate

| **S.No.** | **Sample**  **Code** | **Average hardness** | | **Average disintegration time** | | **% Friability** | |
| --- | --- | --- | --- | --- | --- | --- | --- |
|  |  | **(N) (±STD)** | **Conclusion** | **(Min) ± (STD)** | **Conclusion** | **%** | **Conclusion** |
| 1 | ED-09 | 58.3 ± 15.2 | Passed | 4.17 ± 1.17 | Passed | 0.576 | Passed |
| 2 | EW-01 | 56.96 ± 8.3 | Passed | 3.33 ± 0.52 | Passed | 0.126 | Passed |
| 3 | EG-02 | **26.9 ± 5.8** | **Failed** | 1.33 ± 0.52 | Passed | **Broken** (5.07%) | **Failed** |
| 4 | ED-11 | **29.92 ± 4.4** | **Failed** | 1.17 ± 0.41 | Passed | 0.074 | Passed |
| 5 | ED-02 | 62.7. ± 7.7 | Passed | 2.33 ± 0.52 | Passed | 0 | Passed |
| 6 | EYC-01 | 43.2 ± 8.2 | Passed | 1.17 ± 0.41 | Passed | 0 | Passed |
| 7 | EYC-01’1 | 58.4 ± 6.4 | Passed | 2.17 ± 0.41 | Passed | 0 | Passed |
| 8 | EYCG-01 | 84.8 ± 8.2 | Passed | 3.00 ± 0.63 | Passed | 0 | Passed |
| 9 | ED-08 | 95.5 ± 8.3 | Passed | 2.17 ± 0.41 | Passed | 0.125 | Passed |
| 10 | EDG-01 | **30.7 ± 8.3** | **Failed** | 1.33 ± 0.52 | Passed | 0.678 | Passed |
| 11 | EGG-01 | **33.3 ± 4.6** | **Failed** | 1.50 ± 0.55 | Passed | 0.149 | Passed |
| 12 | EYG-01 | **32.8 ± 3.3** | **Failed** | 3.33 ± 1.51 | Passed | **Broken** | **Failed** |
| 13 | EM-09 | 93.4 ± 7.2 | Passed | 4.00 ± 1.51 | Passed | 0.377 | Passed |
| 14 | EM-07 | 40.3 ± 4.6 | Passed | 5.17 ± 0.41 | Passed | 0 | Passed |
| 15 | EY-04 | **36.3 ± 4.8** | **Failed** | 2.5 ± 0.55 | Passed | 0 | Passed |
| 16 | EY-06 | **30.2 ± 6.2** | **Failed** | 2.17 ± 1.47 | Passed | 0 | Passed |
| 17 | EMG-01 | 86.0 ± 14.6 | Passed | 3.50 ± 0.55 | Passed | 0 | Passed |
| 18 | EY-05 | 59.5 ± 8.6 | Passed | 7.33 ± 0.52 | Passed | 0.695 | Passed |
| 19 | EM-03 | 95.3 ± 11.2 | Passed | 4.17 ± 0.41 | Passed | 0.603 | Passed |
| 20 | EY-01 | **35.0 ± 3.3** | **Failed** | 1.17 ± 0.41 | Passed | 0 | Passed |
| 21 | EM-08 | 93.8 ± 9.5 | Passed | 4.67 ± 0.52 | Passed | **Broken** | **Failed** |
| 22 | EM-08’1 | 65.8 ± 13.7 | Passed | 2.33 ± 1.37 | Passed | 0.088 | Passed |
| 23 | EM-10 | 104.7 ± 12.3 | Passed | 6.50 ± 0.55 | Passed | Coated | Passed |
| 24 | EM-04 | **32.8 ± 4.1** | **Failed** | 1.83 ± 0.41 | Passed | 0.581 | Passed |
| 25 | EM-10’1 | 94.4 ± 9.6 | Passed | 7.33 ± 3.01 | Passed | Coated | Passed |

* SD=Standard Deviation, N=Newton, Min=Minute, mm=Millimeter
