# Supplementary material for: Quality of medicines for Cardio-Vascular Diseases (CVDs) in the Ethiopian border with Kenya: The case of enalapril maleate and furosemide tablet quality in Borena and Gedeo zones
Source: PLOS Glob Public Health. 2024 Jul 15;4(7):e0003104. doi: 10.1371/journal.pgph.0003104 (PMC11249254; doi:10.1371/journal.pgph.0003104)
Supplement: S11 File — (DOC) [file pgph.0003104.s014.doc]

S11 File. Hardness, friability and disintegration test results of furosemide tablets

| **S.No** | **Sample**  **Code** | **Average hardness** | | **Average disintegration time** | | **% Friability** | |
| --- | --- | --- | --- | --- | --- | --- | --- |
|  |  | **(N) (±SD)** | **Conclusion** | **(Min) ± SD** | **Conclusion** | **%** | **Conclusion** |
| 1 | FMG-01 | 90.5 ± 7.9 | Passed | 7.67 ± 1.17 | Passed | 0.125 | Passed |
| 2 | FG-03 | **31.4 ± 2.5** | **Failed** | 2.50 ± 0.55 | Passed | 0.396 | Passed |
| 3 | FDG-01 | 48.6 ± 4.0 | Passed | 1.33 ± 0.52 | Passed | **Broken** | **Failed** |
| 4 | FD-01 | 60.8 ± 14.7 | Passed | 7.50 ± 2.26 | Passed | 0 | **Failed** |
| 5 | FYC-01 | 62.2 ± 9.8 | Passed | 10.3 ± 3.67 | Passed | 0.217 | Passed |
| 6 | FYCG-01 | **13.8 ± 2.6** | **Failed** | 2.33 ± 0.52 | Passed | **Broken** | **Failed** |
| 7 | FD-04 | 56.2 ± 5.9 | Passed | 1.33 ± 0.52 | Passed | **5.268** | **Failed** |
| 8 | FYC-02 | 51.5 ± 9.2 | Passed | 11.67 ± 2.25 | Passed | 0.029 | Passed |
| 9 | FD-10 | **19.9 ± 6.5** | **Failed** | 6.50 ± 0.84 | Passed | **1.940** | **Failed** |
| 10 | FG-02 | **38.1 ± 8.4** | **Failed** | 10.17 ± 1.23 | Passed | 0.524 | **Failed** |
| 11 | FGG-01 | **20.7 ± 6.0** | **Failed** | 2.67 ± 0.82 | Passed | **Broken** | **Failed** |
| 12 | FD-03 | **16.6 ± 2.0** | **Failed** | 1.33 ± 0.52 | Passed | **1.022** | **Failed** |
| 13 | FDG-02 | 45.1 ± 4.7 | Passed | 1.67 ± 0.82 | Passed | 0.452 | Passed |
| 14 | FD-11 | **24.7 ± 3.2** | **Failed** | 3.67 ± 0.82 | Passed | 0.888 | Passed |
| 15 | FM-06 | 45.1 ± 7.8 | Passed | 2.33 ± 1.03 | Passed | 0.146 | Passed |
| 16 | FY-02 | **21.1 ± 3.8** | **Failed** | 2.33 ± 0.52 | Passed | **Broken** | Passed |
| 17 | FM-07 | **29.2 ± 4.3** | **Failed** | 4.67 ± 1.51 | Passed | **Broken** | Passed |
| 18 | FM-01 | **22.7 ± 4.6** | **Failed** | 2.83 ± 0.41 | Passed | 0.087 | Passed |
| 19 | FM-10 | 72.3 ± 12.0 | Passed | 4.33 ± 0.52 | Passed | 0.370 | Passed |
| 20 | FDG-01 | **39.2 ± 1.8** | **Failed** | 1.33 ± 0.52 | Passed | 0.550 | Passed |
| 21 | FM-02 | **9.0 ± 1.9** | **Failed** | 1.67 ± 0.82 | Passed | **Broken** | **Failed** |
| 22 | FYC-03 | **20.1 ± 2.8** | **Failed** | 2.67 ± 0.82 | Passed | 0.784 | Passed |
| 23 | FD-10 | **23.0 ± 2.0** | **Failed** | 3.33 ± 1.03 | Passed | 0.085 | Passed |
| 24 | FY-03 | **23.7 ± 4.1** | **Failed** | 6.17 ± 0.41 | Passed | **1.005** | **Failed** |
| 25 | FM-05 | 49.79 ± 3.8 | Passed | 4.00 ± 0.89 | Passed | 0.461 | Passed |
| 26 | FM-05’1 | 65.5 ± 1.5 | Passed | 4.50 ± 0.55 | Passed | 0 | Passed |
| 27 | FG-01 | **28.7 ± 3.4** | **Failed** | 2.17 ± 0.75 | Passed | 0.592 | Passed |
| 28 | FYG-01 | **26.9 ± 6.1** | **Failed** | 2.33 ± 0.82 | Passed | 0.673 | Passed |
| 29 | FW-01 | 64.8 ± 9.9 | Passed | 7.67 ± 1.21 | Passed | 0.029 | Passed |
| 30 | FD-12 | 57.8 ± 12.5 | Passed | 8.17 ± 1.17 | Passed | 0.294 | Passed |
