# Supplementary material for: Quality of medicines for Cardio-Vascular Diseases (CVDs) in the Ethiopian border with Kenya: The case of enalapril maleate and furosemide tablet quality in Borena and Gedeo zones
Source: PLOS Glob Public Health. 2024 Jul 15;4(7):e0003104. doi: 10.1371/journal.pgph.0003104 (PMC11249254; doi:10.1371/journal.pgph.0003104)
Supplement: S12 File — (DOC) [file pgph.0003104.s015.doc]

S12 File. Uniformity of dosage units of enalapril maleate by content uniformity

| **S.No** | **Sample code (Brand)** | **M** | **X** | **S** | **K** | **M-X** | **|M - X|** | **KS** | **AV=|M - X|+ks** |
| --- | --- | --- | --- | --- | --- | --- | --- | --- | --- |
| 1 | EM-04 (Acepril) | 98.5 | 93.0 | 5.8 | 2.4 | 5.5 | 5.5 | 13.92 | **19.42** |
| 2 | EY-01 (Enali-SSP) | 98.5 | 100.3 | 3.7 | 2.4 | -1.8 | 1.8 | 8.9 | 10.7 |
| 3 | EYC-01 (Envas) | 98.5 | 93.3 | 3.5 | 2.4 | 5.2 | 5.2 | 8.4 | 13.6 |
| 4 | EDG-01 (Enali-SSP) | 98.5 | 96.4 | 2.0 | 2.4 | 2.1 | 2.1 | 4.8 | 7.0 |
| 5 | EM-08 (Encardil) | 98.1 | 97.5 | 0.9 | 2.4 | 0.61 | 0.61 | 2.16 | 2.77 |
| 6 | ED-11 (Enaril) | 98.5 | 87.8 | 2.3 | 2.4 | 10.7 | 10.7 | 5.52 | **16.3** |
| 7 | EYG-01 (Enali-SSP) | 98.1 | 96.8 | 2.1 | 2.4 | 1.3 | 1.3 | 5.04 | 6.34 |
| 8 | EY-05 (Korandil) | 98.5 | 95.8 | 4.8 | 2.4 | 2.7 | 2.7 | 11.52 | 14.22 |
| 9 | EM-10 (Enaril) | 98.5 | 94.0 | 1.7 | 2.4 | 4.5 | 4.5 | 4.08 | 8.6 |

M=Reference value, X=Mean, s=standard deviation, k=acceptability constant, AV= Acceptance value.
