# Supplementary material for: Quality of medicines for Cardio-Vascular Diseases (CVDs) in the Ethiopian border with Kenya: The case of enalapril maleate and furosemide tablet quality in Borena and Gedeo zones
Source: PLOS Glob Public Health. 2024 Jul 15;4(7):e0003104. doi: 10.1371/journal.pgph.0003104 (PMC11249254; doi:10.1371/journal.pgph.0003104)
Supplement: S13 File — (DOC) [file pgph.0003104.s016.doc]

S13 File. Uniformity of dosage units of furosemide by weight variation (n = 3)

| **S.No** | **Brand name** | **Batch No.** | **Sample site** | **Sample code** | **Average weight (mg) ± SD** | **% Deviation (Range)** | **% RSD** |
| --- | --- | --- | --- | --- | --- | --- | --- |
| 1 | Fusid | 31605 | Moyale* | FMG-01 | 199.6 ± 2.68 | (-2.30, 2.20) | 1.34 |
| 2 | Fusix | 1060453 | Gedeb | FG-03 | 113.6 ± 4.08 | (-6.65, 7.44) | 3.60 |
| 3 | Furosemide | LJ5559 | Dilla*** | FDG-01 | 166.4 ± 4.45 | (-6.85, 3.97) | 2.68 |
| 4 | Rasitol | FC002 | Dilla | FD-01 | 158.85 ± 3.54 | (-4.94, 3.87) | 2.23 |
| 5 | Rasitol | FC002 | Yirgachefe | FYC-01 | 162.15 ± 4.22 | (-6.88, 6.07) | 2.60 |
| 6 | Fusix | 1070013 | Yirgachefe* | FYCG-01 | 115.55 ± 2.98 | (-6.53, 4.72) | 2.58 |
| 7 | Furo-Denk | 9ZP | Dilla | FD-04 | 99.5 ± 1.36 | (-1.51, 2.5) | 1.36 |
| 8 | Fruz | BPL763 | Yirgachefe | FYC-02 | 172.7 ± 3.17 | (-3.27, 3.68 ) | 1.83 |
| 9 | Fusix | 1060513 | Dilla | FD-10 | 116.25 ± 4.52 | **(-10.54, 7.53)** | 3.89 |
| 10 | Rasitol | FG004 | Gedeb | FG-02 | 162.7 ± 2.62 | (-3.5, 2.64) | 1.61 |
| 11 | Fusix | 1070063 | Gedeb* | FGG-01 | 115.0 ± 4.89 | (-7.79, 7.87) | 4.26 |
| 12 | Fusix | 1070023 | Dilla | FD-03 | 117.3 ± 4.96 | (-7.08, 9.97) | 4.23 |
| 13 | Furosemide | LJ5560 | Dilla*** | FDG-02 | 165.8 ± 2.10 | (-3.47, 1.96) | 1.27 |
| 14 | Fusix | 1070203 | Dilla | FD-11 | 117.6 ± 5.68 | (-8.97, 8.89) | 4.83 |
| 15 | Furosemide | 210110 | Moyale | FM-06 | 172.8 ± 2.88 | (-2.78, 3.59) | 1.66 |
| 16 | Fusix | 1060373 | Yabelo | FY-02 | 115.7 ± 5.70 | **(-6.61, 10.68)** | 4.93 |
| 17 | Fruz | BPL818 | Moyale | FM-07 | 175.4 ± 4.85 | (-3.62, 4.93) | 2.77 |
| 18 | Fusix | 1060493 | Moyale | FM-01 | 115.4 ± 1.93 | (-2.08, 3.12) | 1.67 |
| 19 | Lefrusid | 78576 | Moyale | FM-10 | 135.7 ± 5.29 | (-8.62, 7.59) | 3.90 |
| 20 | Furosemide | LJ5561 | Dilla*** | FDG-01 | 163.7 ± 3.80 | (-4.67, 4.49) | 2.32 |
| 21 | Frusemide | 2105115 | Moyale | FM-02 | 161.7 ± 22.47 | (-2.82, 3.18) | 1.91 |
| 22 | Fusix | 1060413 | Yirgachefe | FYC-03 | 115.3 ± 3.36 | (-5.46, 6.68) | 2.91 |
| 23 | Fusix | 1060513 | Dilla | FD-10 | 117.5 ± 3.10 | (-4.68, 4.68) | 2.61 |
| 24 | Fusix | 1060423 | Yabelo | FY-03 | 113.7 ± 4.36 | (-6.73,7.35) | 3.83 |
| 25 | Furosemide | 71225 | Moyale | FM-05 | 173.6 ±1.61 | (-1.47, 1.99) | 0.93 |
| 26 | Furosemide | 90456 | Moyale | FM-05’1 | 172.0 ± 2.25 | (-2.33, 2.33) | 1.31 |
| 27 | Fusix | 1060353 | Gedeb | FG-01 | 118.6 ± 3.28 | (-3.88, 6.24) | 2.77 |
| 28 | Fusix | 1070103 | Yirgachefe* | FYG-01 | 118.6 ± 4.56 | (-7.21, 7.13) | 3.85 |
| 29 | Fruz | BPL763 | Wenago | FW-01 | 172.4 ± 4.49 | (-6.01, 4.44) | 2.60 |
| 30 | Fruz | BPL763 | Dilla | FD-09’2 | 170.2 ± 5.94 | (-5.97, 6.38) | 3.49 |

*= Primary Hospital, **= General Hospital, ***= Referral Hospital n= no of replicate
