# Supplementary material for: Quality of medicines for Cardio-Vascular Diseases (CVDs) in the Ethiopian border with Kenya: The case of enalapril maleate and furosemide tablet quality in Borena and Gedeo zones
Source: PLOS Glob Public Health. 2024 Jul 15;4(7):e0003104. doi: 10.1371/journal.pgph.0003104 (PMC11249254; doi:10.1371/journal.pgph.0003104)
Supplement: S14 File — (DOC) [file pgph.0003104.s017.doc]

S14 File. Percentage of enalapril API released at different sampling times (n=5)

| **Time (minutes)** | **% API release (Mean ± SD)** | | | | | |
| --- | --- | --- | --- | --- | --- | --- |
|  | **Enali-SSP** | **Envas-5** | **Acepril** | **Korandil** | **Enaril-5** | **Encardil** |
| 5 | 94.98 ± 0.76 | 60.93 ± 0.39 | 65.41 ± 0.08 | 97.34±0.83 | 51.52 ± 5.23 | 92.52±0.56 |
| 10 | 94.56 ± 1.16 | 87.71 ± 4.38 | 92.91 ±2.45 | 98.56±0.55 | 64.02 ± 3.38 | 96.55±0.46 |
| 15 | 95.56 ± 1.03 | 98.65 ± 0.70 | 91.28±1.79 | 99.20±0.08 | 71.13 ± 2.35 | 96.99±1.26 |
| 30 | 95.56 ± 1.10 | 100.17 ± 1.38 | 92.82±0.19 | 99.18±0.53 | 84.00 ± 0.99 | 98.48±2.15 |
| 60 | 97.27 ± 1.74 | 98.60 ± 2.60 | 91.94±1.45 | 100.01±1.40 | 85.23 ± 0.25 | 99.46±1.02 |
