# Supplementary material for: Quality of medicines for Cardio-Vascular Diseases (CVDs) in the Ethiopian border with Kenya: The case of enalapril maleate and furosemide tablet quality in Borena and Gedeo zones
Source: PLOS Glob Public Health. 2024 Jul 15;4(7):e0003104. doi: 10.1371/journal.pgph.0003104 (PMC11249254; doi:10.1371/journal.pgph.0003104)
Supplement: S15 File — (DOC) [file pgph.0003104.s018.doc]

S15 File. Percentage of furosemide API released at different sampling times (n=6)

| **Sampling time (minutes)** | **% API release (Mean ± SD)** | | | | | | |
| --- | --- | --- | --- | --- | --- | --- | --- |
|  | **Fusix** | **Fusid** | **Fruz** | **Furo-Denk** | **Furosemide** | **Rasitol** | **Leprusid** |
| 5 | 52.22 ± 1.39 | 46.92 ± 9.85 | 19.16 ± 8.65 | 85.56 ± 4.51 | 54.65 ± 4.38 | 54.12 ± 2.56 | 59.88 ± 3.32 |
| 15 | 81.52 ± 0.75 | 75.59 ± 7.44 | 51.91 ± 6.67 | 89.29 ± 3.79 | 73.89 ± 4.26 | 79.38 ± 3.89 | 72.87 ± 10.07 |
| 30 | 84.95 ± 0.68 | 82.47 ± 3.45 | 73.15 ± 2.90 | 87.92 ± 2.54 | 83.29 ± 1.98 | 81.48 ± 3.05 | 77.42 ± 2.49 |
| 45 | 85.28 ± 0.93 | 81.63 ± 1.90 | 78.53 ± 2.71 | 89.44 ± 2.31 | 85.35 ± 1.79 | 82.47 ± 0.76 | 81.26 ± 3.86 |
| 60 | 84.73 ± 1.27 | 83.38 ± 2.49 | 82.59 ± 2.09 | 87.36 ± 1.47 | 86.25 ± 1.49 | 81.09 ± 0.82 | 88.54 ± 4.12 |
| 75 | 86.57 ± 1.78 | 85.97 ± 8.07 | 83.2 ± 2.19 | 88.23 ± 1.44 | 83.85 ± 1.61 | 80.13 ± 0.59 | 89.40 ± 1.91 |

.
