# Supplementary material for: Quality of medicines for Cardio-Vascular Diseases (CVDs) in the Ethiopian border with Kenya: The case of enalapril maleate and furosemide tablet quality in Borena and Gedeo zones
Source: PLOS Glob Public Health. 2024 Jul 15;4(7):e0003104. doi: 10.1371/journal.pgph.0003104 (PMC11249254; doi:10.1371/journal.pgph.0003104)
Supplement: S16 File — (DOC) [file pgph.0003104.s019.doc]

S16 File. Results of TAMC and TYMC on SCDA dishes

## TAMC, TYMC and *E.coli*

Number of CFU/g or CFU/ml = Average number of colonies on 2 SCDA plates × 10 (Dilution Factor)

- In these test, no organisms were recovered from the 2 plates; and the result were
- ＜1 CFU**/**gm (Since there were no colonies formed), so the sample passed according to the USP <62>.
- All negative controls were negative.

Following incubation, the colonies on each sabourand dextrose agar (SDA) plate were counted, and the number of CFU/g or CFU/ml was calculated using the same formula as in TAMC. Therefore, the CFU/gm was less than one, so the sample passed according to USP <62> specification. Table 15 explains that no organisms were recovered from each plate.

The TYMC was considered equal to the number of all colonies forming units found on the SDA plates; if colonies of bacteria were detected on this media, they were counted as part of the TYMC. Similarly, all negative controls were negative.

S16File. Results of TAMC and TYMC on SCDA dishes

| **Incubation end dishes and time** | **Results** | | | | |
| --- | --- | --- | --- | --- | --- |
| TAMC dishes at 21/06/2022  TYMC dishes at 24/06/2022 | | Di Dish 1/cfu | Dish 2/cfu | Average/cfu | cfu/gm | Negative control | | --- | --- | --- | --- | --- | | | | | |
|  | < 1cfu < 1cfu < 1cfu < 1cfu/gm **+** **–**  **✓** | | | | |
|  | < 1cfu | < 1cfu | < 1cfu | < 1cfu/gm | **+** **–**  **✓** |
|  |  |  |  |  |  |
