# Supplementary material for: Quality of medicines for Cardio-Vascular Diseases (CVDs) in the Ethiopian border with Kenya: The case of enalapril maleate and furosemide tablet quality in Borena and Gedeo zones
Source: PLOS Glob Public Health. 2024 Jul 15;4(7):e0003104. doi: 10.1371/journal.pgph.0003104 (PMC11249254; doi:10.1371/journal.pgph.0003104)
Supplement: Attachment — Submitted filename: PGPH-D-23-02514_reviewer.pdf [file pgph.0003104.s020.pdf]

PLOS Global Public Health

Quality of Medicines for Cardio-Vascular Diseases (CVDs) in the Ethiopian Border with Kenya: The Case of Enalapril Maleate and Furosemide Tablet Quality in Borena and Gedeo Zones.

--Manuscript Draft--

**Manuscript Number:**	PGPH-D-23-02514
**Article Type:**	Research Article
**Full Title:**	Quality of Medicines for Cardio-Vascular Diseases (CVDs) in the Ethiopian Border with Kenya: The Case of Enalapril Maleate and Furosemide Tablet Quality in Borena and Gedeo Zones.
**Short Title:**	Quality of cardiovascular drugs in Ethiopia-Kenya border...
**Corresponding Author:**	Ayenew Ashenef<br>Addis Ababa University<br>Addis Ababa, ETHIOPIA
**Order of Authors:**	Esayas Tesfaye
	Fisseha Woldegiorgis
	Tadele Eticha
	Ayenew Ashenef
**Keywords:**	Enalapril maleate, Furosemide, In vitro, Medicine quality, Quality control parameters
**Abstract:**	Background: Hypertension (HTN), a cardiovascular disease (CV), is a major public health challenge. Therefore, the quality of drugs used to treat it has become a major concern. Enalapril and furosemide are among the widely prescribed antihypertensive drugs. This study is aimed at evaluating the quality of different marketed brands of enalapril and furosemide tablets available in the Gedeo and Borena zones, southern Ethiopia.<br>Methods: In this study, thirteen generic brands of enalapril maleate (5 mg) and furosemide (40 mg) tablets were evaluated for visual defects, in vitro dissolution test, weight variations, friability, hardness, and disintegration tests. The analysis of active pharmaceutical ingredient (API) content was performed by high performance liquid chromatography (HPLC).<br>Results: Out of 55 samples, 7 (12.73%) failed to comply with the criteria for visual inspection; otherwise, all samples passed the identification test. Except for one brands of each of enalapril maleate and furosemide, all passed the dissolution test. The assay value showed that all enalapril maleate samples were within the limits of USP (2020). Additionally, except for two batches, all furosemide samples were within the USP and BP (2020) standards. Out of 55 samples, 8% (2/25) and 6.67% (2/30) of enalapril maleate and furosemide tablets failed the uniformity of dosage units (USP-2020) test, respectively. All samples passed the disintegration test, and selected furosemide samples passed the microbial limit tests. However, 36% (9/25) and 20% (6/30) of enalapril maleate and furosemide samples failed to pass hardness test. Generally, from the total sample, 50.91% (28/55) were substandard (did not meet the specification failing any one or more parameters assessed).<br>Conclusions: The studied drugs circulating in the market did not meet some of the needed quality specifications. This could have brought a risk of reduced efficacy due to the distribution of poor quality drugs in the study area.
**Additional Information:**	
**Question**	**Response**
**Financial Disclosure**<br><br>Enter a financial disclosure statement that	The study was sponsored by the Addis Ababa University Graduate research support program to ET and the Ministry of Innovation and Technology, government of Ethiopia, by the project scheme National Problem Solving award to AA.

Powered by Editorial Manager® and ProduXion Manager® from Aries Systems Corporation

describes the sources of funding for the work included in this submission and the role the funder(s) played. This includes grants and any commercial funding of the work or authors.<br><br>This statement will be typeset if the manuscript is accepted for publication.<br><br>*Please review the submission guidelines and the instructions link below for detailed requirements and guidance.*	
**Competing Interests**<br><br>On behalf of all authors, disclose any competing interests that could be perceived to bias this work.<br><br>This statement will be typeset if the manuscript is accepted for publication.<br><br>*Please review the instructions link below and PLOS Global Public Health's competing interests policy to determine what information must be disclosed at submission.*	The authors declare that they did not have any conflicts of interest to declare in relation to this study and publication. The funders did not have any role in the study design, data interpretation or publication.
**Data Availability**<br><br>Before publication, Authors are required to make fully available and without restriction all data underlying their findings. Please see our PLOS Data Policy page for detailed information on this policy.<br><br>A **Data Availability Statement**, detailing where the data can be accessed, is required at first submission. Insert your Data Availability Statement in the box below.<br><br>Please see the data reporting section of	The datasets used in this publication are available upon reasonable request from the corresponding author

our submission guidelines for instructions on what you need to include in your Data Availability Statement.<br><br>This statement will be typeset if the manuscript is accepted for publication.<br><br>*PLOS allows rare exemptions to address legal and ethical concerns. If you have legal or ethical restrictions, please use the box below to detail these in full sentences for the Journal team to consider.*	

Powered by Editorial Manager® and ProduXion Manager® from Aries Systems Corporation

Manuscript

Click here to access/download;Manuscript;Cardiovascular drugs quality Ethiopia-Kenyaok.doc

Quality of Medicines for Cardio-Vascular Diseases (CVDs) in the Ethiopian Border with Kenya: The Case of Enalapril Maleate and Furosemide Tablet Quality in Borena and Gedeo Zones.

Esayas Tesfaye[1, 3], Fisseha Woldegiorgis [2], Tadele Eticha[1], Ayenew Ashenef[1,4*]

[1]Department of Pharmaceutical Chemistry and Pharmacognosy, School of Pharmacy, College of Health Sciences, Addis Ababa University, P.O. Box. 1176, Addis Ababa, Ethiopia

[2]Humanwell Pharmaceutical Ethiopia PLC, North Shoa, Tulefa, Ethiopia

[3]Department of Pharmacy, College of Health Science, Dilla University, P.O. Box. 419, Dilla, Ethiopia.

[4]Center for Innovative Drug Development and Therapeutic Trials for Africa (CDT-Africa), College of Health Sciences, Addis Ababa University, P.O. Box. 9086, Addis Ababa, Ethiopia

*Corresponding author P.O. Box: 1176, Addis Ababa University, Ethiopia, E-mail Address: ayenew.ashenef@aau.edu.et

ET: Esayas Tesfaye, esa.tesfaye7@gmail.com

FW: Fisseha Woldegiorgis, fishergaru@gmail.com

TE: Tadele Eticha, td.eticha@gmail.com

AA: Ayenew Ashenef, ayenew.ashenef@aau.edu.et

Abstract

Background: Hypertension (HTN), a cardiovascular disease (CV), is a major public health challenge. Therefore, the quality of drugs used to treat it has become a major concern. Enalapril and furosemide are among the widely prescribed antihypertensive drugs. This study is aimed at evaluating the quality of different marketed brands of enalapril and furosemide tablets available in the Gedeo and Borena zones, southern Ethiopia.

Methods: In this study, thirteen generic brands of enalapril maleate (5 mg) and furosemide (40 mg) tablets were evaluated for visual defects, *in vitro* dissolution test, weight variations, friability, hardness, and disintegration tests. The analysis of active pharmaceutical ingredient (API) content was performed by high performance liquid chromatography (HPLC).

Results: Out of 55 samples, 7 (12.73%) failed to comply with the criteria for visual inspection; otherwise, all samples passed the identification test. Except for one brands of each of enalapril maleate and furosemide, all passed the dissolution test. The assay value showed that all enalapril maleate samples were within the limits of USP (2020). Additionally, except for two batches, all furosemide samples were within the USP and BP (2020) standards. Out of 55 samples, 8% (2/25) and 6.67% (2/30) of enalapril maleate and furosemide tablets failed the uniformity of dosage units (USP-2020) test, respectively. All samples passed the disintegration test, and selected furosemide samples passed the microbial limit tests. However, 36% (9/25) and 20% (6/30) of enalapril maleate and furosemide samples failed to pass hardness test. Generally, from the total sample, 50.91% (28/55) were substandard (did not meet the specification failing any one or more parameters assessed).

Conclusions: The studied drugs circulating in the market did not meet some of the needed quality specifications. This could have brought a risk of reduced efficacy due to the distribution of poor quality drugs in the study area.

Keywords: Enalapril maleate, Furosemide, *In vitro*, Medicine quality, Quality control parameters

Background

Cardiovascular disease is the term used to describe diseases affecting the heart and circulatory system, including stroke and raised blood pressure (hypertension) [1]. In 2017, the American College of Cardiology/American Heart Association Task Force on Clinical Practice Guidelines redefined hypertension in adults as systolic blood pressure ≥ 130 mmHg and/or diastolic blood pressure ≥ 80 mmHg [2]. Most patients with hypertension (HTN) require drug treatment to achieve sustained reduction of blood pressure. Loop diuretics or high-ceiling, β-blockers, calcium antagonists, angiotensin converting enzyme inhibitors (ACE-Is) and angiotensin II receptor blockers (ARBs) can adequately lower blood pressure and reduce the risk of cardiovascular (CV) death and morbidity [3]. Thus, these drugs are all recommended for the initiation and maintenance of blood pressure control, either as monotherapy or in combination for an additive or synergistic effect.

Cross border trade between Ethiopia and Kenya is an important feature that is existing for many years. Informal cross-border trade between Ethiopia and Kenya is substantial and vital for both countries. [4]. Our study area Moyale and Mendera are the two main trading hubs across the border between Kenya and Ethiopia. Ethiopia's main exports include livestock, livestock products and cereals. On the other hand, Kenya's exports to Ethiopia are manufactured products including processed food. Similarly cross-border trade in medicine in the borderlands of Ethiopia and Kenya exists but not as such recorded adequately [5]. Hence we are assessing the selected medicines quality that may enter to Ethiopia through informal means without the national medicine authority (NMA) regulatory scrutiny process on medicine importation.

This study was focused on enalapril maleate (angiotensin converting enzyme inhibitor) and furosemide (diuretics), which are very common and widely prescribed in the management of HTN [6,7].

Enalapril maleate is used to prevent, treat or improve the symptoms of hypertension. It is also used in the management of symptomatic heart failure or asymptomatic left ventricular dysfunction, coronary artery disease, and certain chronic kidney diseases, with a favorable efficacy and tolerability profile [8]. It is a prodrug that is bioactivated by hydrolysis of the ethyl ester to enalaprilat, which is the active angiotensin converting enzyme inhibitor. Inhibition of ACE results in decreased plasma angiotensin II, which leads to decreased vasopressor activity aldosterone secretion [9]. Chemically described as (S)-1-[N-[1-(ethoxycarbonyl)-3-phenylpropyl]-L-alanyl]-L-proline, (Z)-2 butenedioate salt (1:1) with the empirical formula $C_{20}OH_{28}N_2O_5$• $C_4H_4O_4$ [10]. The structural formula of enalapril maleate is given in Figure 1(A). It is a white to off-white, crystalline powder with a molecular weight of 492.53 [11]. The WHO only lists strengths of 2.5 and 5.0 mg in their list of essential drugs [12].

Furosemide is the most commonly used loop diuretic (furosemide, bumetanide, torsemide and ethacrynic acid) [9]. These drugs have the highest efficacy in mobilizing Na^+ and Cl^- from the body. It inhibits the cotransport of Na+/K+/2Cl– in the luminal membrane in the ascending limb of the loop of Henle [13]. Chemically, it is 5 (aminosulfonyl)-4-chloro-2-[(2-furanylmethyl)-amino] benzoic acid. It is a white to slightly yellow, odorless, crystalline powder with a molecular weight of 330.77 [14]. Its empirical formula is $C_{12}H_{11}ClN_2O_5S$, and the structural formula is given in Figure 1(B) (12)(13). The bioavailability of furosemide from oral dosage forms is highly variable due to its poor solubility. Thus the difference in formulation aspects

from brand to brand as a result of the manufacturer’s technicalities will make their pharmacokinetic profile prone to high variability in such class of medicines[15].

Figure 1 uploaded here

Several studies have been carried out on the quality control of enalapril maleate and furosemide tablets in some countries across the globe. In a study conducted in India, most of the enalapril maleate brands passed except for variation in hardness, disintegration time and dissolution profile during the test procedure [16]. Another study conducted in Brazil and Guatemala revealed that all samples of enalapril tablets showed satisfactory results in pharmacopoeial specification. In Turkey, study samples complied with the pharmacopoeia standards except hardness and friability properties [17,18,19]. A study conducted in Brazil and Argentina also revealed that all brands of furosemide tablets passed concerning dissolution profiles [20,21,22].

In a study conducted in Nigeria, except for a few samples that failed in hardness, most of the furosemide tablet batches tested met with label claims in terms of the content of furosemide and dissolution profile [23]. A similar study in 10 sub-Saharan countries revealed that the prevalence of poor-quality drugs differed significantly between drugs, including a 12.5% substandard and falsified (SF) prevalence for furosemide. The proportion of poor-quality drugs exceeded 20% in other African countries (24). However, a similar study in Libya revealed that all brands were in compliance with the specifications [7].

A study conducted in the Ethiopia capital city Addis Ababa showed that the majority of furosemide brands passed quality attributes but failed in hardness and in vitro drug dissolution

profiles [25]. However, a study performed in Bahir Dar city, Northwest Ethiopia, revealed that all furosemide brands met all quality specifications per the pharmacopoeial specifications [26].

The aim of this study was to evaluate the quality of enalapril and furosemide samples collected from southern Ethiopia, specifically from the Gedeo and Borena zones. These areas were targeted due to cross-border illegal trade of medicine exists with the Ethiopian-Kenyan border.

Materials and Methods

Materials

Enalapril maleate and furosemide standards were used that were available at Humanwell Pharmaceutical Company PLC. Methanol for HPLC was purchased from Merck Life Science Private Limited, Germany. Sodium hydroxide pellets (or pearls) 97% Extra Pure were purchased from Loba Chemie Pvt. Ltd. Mumbai, India. Tetrahydrofuran, glacial acetic acid and phosphoric acid (85%) were purchased from sd fine chEM, Loba Chemie Pvt. Ltd., Mumbai and Delhi, India, respectively. Monobasic potassium and sodium phosphate were purchased from Loba Chemie Pvt. Ltd, Mumbai, India. Acetonitrile was purchased from Merck Life Science Pvt Ltd, Germany. Soybean-casein digest agar (SCDA), soybean-casein digest broth (SCDB), Sabouraud dextrose agar (SDA), MacConkey agar (MCA), and MacConkey broth (MCB) were obtained from Beijing, China.

Sampled drug products

All 55 samples of medicines that included enalapril maleate (25/55) and furosemide (30/55) were purchased for quality testing from retail pharmacies and hospitals in Gedeo and Borena Zones,

southern Ethiopia (Sampling sites are shown in figure 2 map), and were within their expiration dates before the test. The details of the two sampled medicines were described in supplementary tables 1 and 2. Samples were collected from 39 drug outlets (as shown on supplementary table3), of which 86.8% of samples were collected from private facilities, whereas the remaining 13.2% were collected from government facilities. The map depicting the sampling sites is given in figure 1 in the per their GPS coordinates. During the sample collection period, six and seven brands of enalapril maleate and furosemide tablets (as described on supplementary table 4) were found in the market, respectively.

{Insert Figure 2}

Inclusion and exclusion criteria

Samples were collected based on inclusion and exclusion criteria. The study was ethically approved by the Addis Ababa University School of Pharmacy Ethical Review Committee/board as ERB/SOP/361/13/2021 dated September 29, 2021. In addition, a letter of cooperation was written from the Ethiopian Food and Drug Authority Moyale entry and exit port branch office in support of the study in the area. The inclusion criteria were to collect all tablet dosage forms of enalapril maleate and furosemide available in a given retail outlet selected for this study. The exclusion criterion was to avoid collection of the same product with a similar batch number at the same sample collection site.

In vitro quality evaluation of enalapril maleate and furosemide

The parameters assessed include visual inspection by the Joint WHO/USP/FIP check list [27], identity tests by USP and BP methods [28,29], assay by pharmacopoeial methods [30], uniformity of dosage units by USP 2020 method, test for hardness, test for friability, disintegration and dissolution tests, and total aerobic microbial count, total yeast and mold count and *E. coli* assessment by pharmacopoeial methods [31].

The laboratory works were performed in the National Medicine regulatory agency (NMRA) current good manufacturing practice (CGMP) certified pharmaceutical industry quality control laboratory per their sops in place to assure all quality assurance procedures are adhered. Thus, the reliability of the data generated is assured to current standards of the industry.

Results

Physical characteristics, packaging and labeling information

According to visual inspection of the WHO, EU and USP checklists, the majority of enalapril maleate and furosemide tablets sampled were well blistered and packed. The packages were appropriately labeled. However, there were seven (12.72%) samples: three from enalapril maleate and four from furosemide with poor packaging and the absence of a leaflet or package insert. Few furosemide samples were cracked, eroded and caked. From a total of 55 tablets, 12% (3/25) of enalapril maleate and 13.33% (4/30) of furosemide tablets had poor physical characteristics and appearances, respectively. Tables 1 and 2 explain failed samples on packaging and physical characteristics. Detailed descriptions and images of failed visual tests were fully shown on the supplementary files in the visual defects section.

Table 1. Physical characteristics of the tablets

Sample (Brand)	Uniformity of colour	Uniformity of size	Uniformity of shape	Surface spot or contamination	Breaks, and cracks
Enalapril (Korandil)	✓	✓	✓	X	X
Enalapril (Enali-SSP)	✓	✓	✓	✓	X
Furosemide (Fusix)	✓	✓	✓	✓	X
Furosemide (Fruz)*	✓	✓	X	X	X
Furosemide (Frusemide)	✓	✓	✓	X	✓

'X'= Failed sample, '✓'= Passed sample

Table 2. Packing and labeling information for the different brands

Product code	Medicine strength (mg/tablet)	Dosage Statement	Batch/Lot no.	Storage condition	Manufacturing date	Expiry date
Enalapril (Korandil)	✓	✓	✓	✓	11/2020	11/2023
Enalapril (Enali-SSP)	✓	✓	✓	✓	11/2020	10/2022
Furosemide (Fusix)	✓	✓	✓	✓	06/21	06/24
Furosemide (Fruz)	✓	✓	✓	✓	04/2021	03/2024
Furosemide (Frusemide)	✓	✓	✓	✓	05/2021	04/2024
Furosemide (Fruz)	✓	✓	✓	✓	11/2020	11/2023

Identification

Identification test results of enalapril by HPLC

The identity of the tested products was confirmed by comparing their retention times with that of the enalapril reference standard. All enalapril samples analyzed displayed retention times corresponding with those of the respective reference standards. Figure 3 depicts one representative enalapril sample code, EM-10, collected from Moyale, which contained the correct API compared to its reference standard enalapril maleate chromatogram.

Figure 3 uploaded here

Identification test results of furosemide by UV

From the total of 30 samples, 20 samples were manufactured in compliance with the USP specification, and thus, an identity test was performed according to USP-43, 2020. The UV absorbance maxima and minima results for the furosemide reference standard were 227.00 nm and 332.42 nm, respectively. Similarly, every sample maxima and minima exhibited very close to the wavelength of the furosemide standard. Figure 4 depicts the identification test of the standard and a representative sample coded FW-01 of furosemide collected from the Wenago site.

Figure 4 uploaded here

Similarly, the remaining 10 samples of furosemide were manufactured by BP specification; therefore, identification tests were performed according to BP 2020. By scanning in the range 220 to 320 nm of the final solution obtained in the assay, the samples exhibit two maxima, at 228

nm and 271 nm. In these tests, the samples exhibit absorption maxima from 226.609 to 228.565 nm and absorption minima from 269.565 to 269.840 nm. There were few wavelength deviations observed in both the absorption maxima and minima. The details obtained in the identification tests for furosemide are included in the supplementary files S. Tables 5 and 6.

Assay

Assay result of enalapril maleate

The assay values for enalapril drug products ranged from 91.35% to 104.53%. This showed that all batches comply with the USP specification limit (90–110%). All assay values are given in supplementary table 7.

System suitability test results for the enalapril maleate assay

Five replicates of 50 µl enalapril maleate USP reference standard solutions were injected into the HPLC system, and the chromatograms were recorded to evaluate the system suitability parameters as the tailing factor (NMT 1.5), theoretical plate number (NLT 300 for enalapril), and % RSD (NMT 2.0). According to the above parameters, these system suitability test criteria were met before the sample analyses.

HPLC analysis of enalapril

For the analysis of the drug, the calibration curve was prepared using 6 concentrations within the range of 0.08 mg/mL to 0.28 mg/mL enalapril. The internal standard was added to each sample, and the peak area ratio was plotted against the concentration (Figure 5). The calibration curve was found to be linear in the calibration range with a regression factor $R^2 = 0.9998$.

Figure 5 uploaded here

Assay result of furosemide

Assay results using BP and USP 2020

From 30 samples, 10 samples were analyzed using the BP method, while for 20 samples, the USP method was employed. The *absorbance* of the resulting solution at the maximum at 271 nm was measured. The minimum and maximum stated content (Assay) of furosemide samples were 92.33% and 101.11%, respectively, and passed the BP specification limit (95–105%). However, two samples from the Fusix brand failed to meet the pharmacopoeial specification. The assay value performed using HPLC for furosemide drug products ranged from 91.68% to 101.22%. These values are in compliance with the USP specification. The detail values for each sample are shown on Supplementary tables 8 and 9.

HPLC system suitability test result of the furosemide standard

Five replicates of 20 μL of furosemide USP reference standard solutions were injected into the HPLC system, and chromatograms were recorded to evaluate the system suitability parameters, such as the tailing factor (NMT 1.5), theoretical plate number (NLT 2000 for furosemide), and % RSD (NMT 2.0). According to the above parameters, the system suitability test data passed the requirements

Calibration curve of the furosemide standard for the assay test

For the analysis of the drug, the calibration curve was prepared using 6 concentrations within the range of 0.4 mg/mL to 1.4 mg/mL furosemide. The concentrations of furosemide RS against the peak area ratio were plotted to obtain the calibration curves. The calibration curve was found to be linear in the calibration range with a regression factor $R^2 = 0.9996$. Figure 6 depicts the

chromatogram of a standard sample and representative furosemide sample code FM-06 collected from Moyale city.

Figure 6 uploaded here

Hardness test

This study revealed that from the total of 55 samples, 36% (9/25) and 20% (6/30) of enalapril maleate and furosemide samples failed the hardness tests, respectively. These failed samples were from the Fusix brand. The maximum and minimum mean hardness test results for enalapril maleate tablets were 104.7 ± 12.3 and 26.9 N ± 5.8, respectively, whereas for the furosemide tablets were 90.5 N ± 7.9 and 9.0 N ± 1.9, respectively.

Disintegration time test

According to this study, all the samples passed the disintegration time test. The maximum mean disintegration time test results for enalapril maleate and furosemide tablets were 7.33 ± 3.01 and 11.67 ± 2.25 minutes, respectively. The maximum mean disintegration time of enalapril maleate was observed for the coated tablet. However, their minimum average disintegration time test results for enalapril maleate and furosemide tablets were 1.17 ± 0.41 minutes and 1.33 ± 0.52 minutes, respectively.

Friability test

In this study, most batches of the Fusix brand failed to comply with the USP specification for the friability test. Of the 55 samples, 12% (3/25) and 33.33% (10/30) of enalapril maleate and furosemide samples, respectively, failed the friability test. The percentage friability test results

for enalapril maleate and furosemide tablets ranged from 0% to 5.07% and from 0% to 5.27%, respectively.

Table 3. Distribution of failed enalapril samples for hardness test across different sampling site.

S.No	Sample code	Brand name	Batch No.	Collection site	Hardness test value (Mean ± SD)	Conclusion
1	EG-02	Enali-SSP	06121060030	Gedeb	26.9 ± 5.8	Failed
2	ED-11	Enaril-5	SDJ662	Dilla	29.92 ± 4.4	Failed
3	EDG-01	Enali-SSP	06121060010	Dilla	30.7 ± 8.3	Failed
4	EGG-01	Enali-SSP	06121070030	Gedeb	33.3 ± 4.6	Failed
5	EYG-01	Enali-SSP	06120110010	Yabelo	32.8 ± 3.3	Failed
6	EY-04	Enali-SSP	06121060010	Dilla	36.3 ± 2.8	Failed
7	EY-06	Enali-SSP	06121070020	Yabelo	30.2 ± 6.2	Failed
8	EY-01	Enali-SSP	061201100110	Yabelo	35.0 ± 3.3	Failed
9	EM-04	Envas-5	D21012BX52	Moyale	32.8 ± 4.1	Failed

SD = Standard Deviation

The brands Enali-SSP and Encardil-5 of enalapril maleate failed the friability test according to the USP specification. Similarly, these failed samples are not registered in the Ethiopian regulatory information system of the Ethiopian Food and Drug Authority (EFDA). Detailed results and the distribution of failed enalapril maleate samples for the friability test across different sampling sites are shown in Table 4.

Table 4. Distribution of failed enalapril maleate samples for % friability test across different sampling site.

S.No	Sample code	Brand name	Batch No.	Collection site	Friability test value	Conclusion
1	EG-02	Enali-SSP	06121060030	Gedeb	Broken	Failed
2	EYG-01	Enali-SSP	06120110010	Yabelo	Broken	Failed
3	EM-08	Encardil-5	D00924	Moyale	Broken	Failed

The brand Fusix of furosemide tablets failed the hardness test according to the specification. These failed drugs were also collected from different sites, as explained in Table 5.

Table 5. Distribution of failed furosemide samples for hardness test across different sampling site.

S.No	Sample code	Brand name	Batch No.	Collection site	Hardness test value	Conclusion
1	FG-03	Fusix	1060453	Gedeb	31.4 ± 2.5	Failed
2	FYCG-01	Fusix	1070013	Yirgachefe	13.8 ± 2.6	Failed
3	FD-10	Fusix	1060513	Dilla	19.9 ± 6.5	Failed
4	FY-03	Fusix	1060423	Yabelo	23.7 ± 4.1	Failed
5	FG-01	Fusix	1060353	Gedeb	28.7 ± 3.4	Failed

6	FYG-01	Fusix	1070103	Yabelo	26.9 ± 6.1	Failed

Different brands of furosemide tablets failed the friability test according to the USP specification. These failed drugs were also collected from different sites, as explained in Table 6.

Table 6. Distribution of failed furosemide samples for % friability test across different sampling site.

**S.No**	**Sample code**	**Brand name**	**Batch No.**	**Collection site**	**% Friability test value**	**Conclusion**
1	FDG-01	Furosemide	LJ5561	Dilla	Broken	Failed
2	FYCG-01	Fusix	1070013	Yirgachefe	Broken	Failed
3	FD-04	Furo-Denk	9ZP	Dilla	5.268	Failed
4	FD-10	Fusix	1060513	Dilla	1.940	Failed
5	FGG-01	Fusix	1070063	Gedeb	Broken	Failed
6	FD-03	Fusix	1070023	Dilla	1.022	Failed
7	FY-02	Fusix	1060373	Yabelo	Broken	Failed

8	FM-07	Fruz	BPL818	Moyale	Broken	Failed
9	FM-02	Furosemide	2105115	Moyale	Broken	Failed
10	FY-03	Fusix	1060423	Yabelo	1.005	Failed

The results of friability, hardness, and disintegration tests for all samples are given on supplementary tables 10 and 11.

Uniformity of dosage units

Content uniformity of Enalapril maleate

For uniformity of dosage units of enalapril, a content uniformity test was performed using a method specified in USP 2020. According to the specification, the requirements for dosage uniformity are met if the acceptance value of the first 10 dosage units is less than or equal to L1%, which is 15.

Content uniformity of Enalapril maleate using HPLC

Five replicates of 50 µl enalapril maleate USP reference standard solutions were injected into the HPLC system, and the chromatograms were recorded to evaluate the system suitability parameters, such as the tailing factor (NMT 1.5), theoretical plate number (NLT 300 for enalapril), and % RSD (NMT 2.0). The RSD was 0.99, the theoretical number of plates was 1596.6, and the tailing factor was 0.88. According to these parameters, the system suitability test data for HPLC were set up before the content uniformity of enalapril measurements. It passed the requirements.

This study showed that 8% (2/25) of enalapril maleate samples collected from Moyale had failed for uniformity of dosage units. Brands of enalapril, ACEPRIL and ENCARDIL (with batch numbers 75034 and D00923) from Moyale failed the content uniformity test, with results of 19.42 and 16.3, respectively. The detail results for all assessed samples are shown on supplementary tables 12 and 13.

Weight variation of furosemide tablets

In this study, even though major samples of furosemide passed, two out of thirty (6.67%) of samples deviated, generating values above and below the pharmacopoeial specification (USP-2020). The failed furosemide of brand FUSIX, with batch numbers 1060513 and 1060373, was collected from Dilla and Yabelo, respectively. All results obtained are shown on supplementary table 13.

Dissolution

Dissolution test of enalapril maleate

Five replicates of 50 μL enalapril maleate USP reference standard solutions were injected into the HPLC system, and the chromatograms were recorded to evaluate the system suitability parameters, such as the tailing factor (NMT 1.5), theoretical plate number (NLT 300 for enalapril), and % RSD (NMT 2.0). The system suitability test data for enalapril were fulfilled.

For the analysis of enalapril maleate, the calibration curve was prepared using 6 concentrations within the range of 0.08 mg/mL to 0.28 mg/mL of enalapril by taking 4, 6, 8, 10, 12, and 14 mL from the stock solution. The regression equation was $y = 22.835x + 25.007$, where y is the peak

response and x is the concentration in mg/mL. With a regression factor of $R^2 = 0.9998$, the calibration curve was found to be linear in the calibration range.

The dissolution profiles of six brands of enalapril maleate passed the USP 43/NF 38 specification limits. As per the specification, enalapril maleate tablets should not release < 80% of the labeled amount within 30 minutes. However, the brand ENARIL-5 with batch number SDJ662, which released an API of less than 80% (77.50 ± 1.91), did not comply with the USP specification. Figure 7 (A) depicts the time-dependent dissolution profiles of all brands of enalapril maleate at different sampling points (5, 10, 15, 30, and 60 minutes). Detail exact values are described in the supplementary table 14.

Dissolution test of furosemide

In the calibration curve, a linear regression equation was $y = 0.0616x - 0.0102$, where y is the absorbance and x is the concentration in μg/mL. The calibration curve showed a linear relationship between the concentration of the tested samples and the absorbance values over the concentration range of 3.2-11.2 μg/mL ($R^2 = 0.9994$).

This study showed that the mean dissolution test results for different brands of furosemide tablets comply with the USP specification. As per the USP 43/NF 33 specification, furosemide tablets should not release < 80% of the labeled amount within 60 minutes. The collected furosemide samples mean percentage of API released at 60 minutes. The values obtained ranged from 81.09 ± 0.82 to 88.54 ± 4.12%. However, one brand of Fruz collected in Moyale city with batch number BPL818 failed to meet the USP specification (46.73 ± 0.66). Figure 7 (B) depicts the time-dependent dissolution profiles of all brands of furosemide at different sampling points (5,

15, 30, 45, 60, and 75 minutes). The exact values of this test had been included in supplementary table 15.

Figure 7 uploaded here

Result and calculation for TAMC, TYMC and *E. coli*

Result and calculations for TAMC

After incubation, the number of colonies was counted from each Soybean-Casein Digest Agar (SCDA) plate. This number of CFU/gm or CFU/mL was calculated as:

Number of CFU/g or CFU/mL = Average number of colonies on 2 SCDA plates × 10 (Dilution Factor)

Since there were no colonies formed in the study, the sample passed according to the USP <62> criteria [31].

Result and calculations for TYMC

After incubation, the numbers of colonies were counted from each Sabourand dextrose agar (SDA) plate. The number of CFU/g or CFU/ml was calculated according to the above formula as in TAMC. In the case of if test organisms were recovered from the 2 plates, and the result as described above as in TAMC. The sample passed according to USP <62> specification. The TYMC was considered to be equal to the number of all Colony Forming Units found on the SDA plates; if colonies of bacteria were detected on this medium, they were counted as part of TYMC. Similarly, all negative controls were negative.

Test for specified microorganisms *(Escherichia coli)*

Enrichment for Escherichia coli (E. coli)

The specified microorganism *Escherichia coli* with MacConkey Agar were tested by enrichment. After four days, the observed result was the absence of any colonies on the streaking lines of the petri dishes. Therefore, it was not detected for a defined unit, and the sample passed the test. The results for the microbiology tests are included in the supplementary files as supplementary table 16 and S. figure 2 as well as 3.

Summary of overall quality assessment results

From this study, 12.73% (7/55) of the total sample failed the visual inspection test, 3.64% (2/55) failed the assay test, 7.27% (4/55) failed the uniformity of dosage units' test, 27.27% (15/55) failed the hardness test, 23.64% (13/55) failed the friability test, and 3.6% (2/55) failed the dissolution test. Of the 55 collected samples, 48% (12/25) of enalapril samples and 53.33% (16/30) of furosemide samples were substandard, failing one or more of the tested parameters. Overall, 50.91% (28/55) of the total collected samples were substandard. Apart from that, all studied samples passed identification and disintegration tests. The details are shown in table 7.

Table 7. Overall quality test results of enalapril and furosemide samples

Product (sample)	Samples failing quality parameters test							
	Visual inspection	Identification	Assay	Uniformity of dosage unit	Hardness	Friability	Disintegration	Dissolution
Enalapril	12% (3/25)	0% (not failed)	0% (not failed)	8% (2/25)	36% (9/25)	12% (3/25)	0% (not failed)	4% (1/25)
Furosemide	13.33% (4/30)	0% (not failed)	6.67% (2/30)	6.67% (2/30)	20% (6/30)	33.33% (10/30)	0% (not failed)	3.33% (1/30
Overall	12.73% (7/55)	0% (not failed)	3.64% (2/55)	7.27% (4/55)	27.27% (15/55)	23.64% (13/55)	0% (not failed)	3.64% (2/55

Discussion

In this study, the pharmaceutical quality of commonly available brands of enalapril maleate and furosemide tablets in the Gedeo and Borena zones of southern Ethiopia was assessed.

In this study, the results of the visual inspection of the analyzed samples showed problems with packaging. Defects in the physical characteristics of the products were also observed. Poor packaging, the absence of leaflets or package inserts and different leaflets exist in different brands. Furthermore, cracked, eroded, and caked samples were observed during visual inspection. Therefore, these failed samples imply that they were not intact as genuine medicine. However, according to a study performed in Brazil, all samples of inspected enalapril tablets showed satisfactory visual inspection, and none of the analyzed tablets were outside the visual inspection tools [17]. In contrast to this study finding, a study conducted in the Ethiopia capital city Addis Ababa [25] and Bahir Dar city [26] also shows that all samples of furosemide passed the visual inspection tools.

In the current study, all brands and batches of enalapril maleate and furosemide tablets assessed for identity passed the USP (2020) and BP (2020) methods. Identification tests performed on the drug in various studies showed the same results. As in the present study, all brands of enalapril maleate analyzed passed an identity test in a study performed in Brazil [17] and Guatemala [18]. Another study in Turkey [19] and Nigeria also reported the presence of API in different brands of enalapril maleate. Similarly, a study conducted in Canada [32], Nigeria [23], Libya [7], Ethiopia at Bahir Dar city [26] showed the presence of the furosemide API.

The findings of the present study revealed that all batches of enalapril maleate met the standard criteria of the specification in terms of the assay of active ingredients. Similarly, all brands of furosemide performed by USP 2020 passed the specification. However, out of ten furosemide samples analyzed by BP 2020, two samples had less than 95% API content. A similar study conducted in Brazil [17], Guatemala [18] and Turkey [19] showed that all samples of enalapril maleate passed the USP pharmacopoeial specification. A similar survey conducted by the USP method in two study areas of Ethiopia, Addis Ababa [25] and Bahir Dar city [26], revealed that all samples of furosemide passed the assay test.

The results of the current study revealed that most samples of both enalapril maleate and furosemide samples fulfilled the acceptance criteria for dosage uniformity. However, 8% (2/25) of enalapril maleate and 6.67% (2/30) of furosemide samples did not meet these pharmacopoeial acceptance criteria (USP, 2020). However, these findings are not similar to those of previous studies performed in India [16], Brazil [17] , Guatemala [18] and southern Nigerian cities [33], in which all the samples passed the dosage uniformity test of enalapril maleate. This study result is also not similar to the studies performed in Canada [32], Nigeria [23], Libya [7] and in Ethiopia

Bahir Dar city [26]. This difference may be related to manufacturing practices. The production processes could be the cause of the difference in content uniformity tests. This might be because variable amounts of excipients and/or API may be used by manufacturers in varied ratios for the drug products, particle density, shape and size of the tablets.

This study revealed that 36% (11/25) and 20% (6/30) of enalapril maleate and furosemide samples failed the hardness tests, respectively. In the present study, the majority of the failed furosemide brands were Fusix. It is below the minimum value of 40 N [34]. This weak hardness results in a crushing strength that also causes the product to disintegrate and dissolve easily. As a result, those failed samples passed the disintegration and dissolution tests easily. At the same time, failed brands of enalapril and furosemide failed the friability test. This association shows that samples with weak hardness have a direct effect on friability. Similarly, during visual inspection, breakage, chipping, and the caked appearance of the sample might also be due to the low crushing strength of the tablet. Some of these enalapril maleate results are in agreement with the study reported in Turkey, in which all the tablets in this study did not possess enough hardness [19], and southern Nigerian cities [33] indicated that most brands of enalapril maleate failed the hardness test. Another study conducted in Nigeria that agreed with the current study showed that few of the furosemide brands had failed the hardness test [23]. Additionally, in a study conducted in the Ethiopia capital city Addis Ababa, some brands of furosemide failed to meet the minimum crushing strength [25]. Failure to meet the specification of the hardness test may be due to looseness of interparticulate bonding or the use of low compression pressure in the tablet machine [34].

In this study, most batches of the Fusix brand failed to comply with the test for friability per the USP 2020 specification. Out of 25 samples, 12% (3/25) and 33.33% (10/30) of enalapril maleate and furosemide samples failed the friability test. The same three batches of the FUSIX brand failed both the hardness and friability tests simultaneously. Similarly, two batches of ENALI-SSP failed both hardness and friability tests. The samples failing both friability and hardness tests might have common failure factors. A study in Turkey agreed with this study in that some enalapril brands were found to contain failed friability tests that were outside the appropriate limits [19]. A similar study conducted in southern Nigerian cities [33] indicated that most brands of enalapril maleate failed the hardness test. In contrast to these study findings, a study conducted in Brazil [17] and the Ethiopia capital city Addis Ababa [25] showed that all tested furosemide samples possess satisfactory results in the friability test.

In the present study, all brands and batches of enalapril maleate and furosemide tablets were disintegrated in less than 15 minutes. The maximum mean disintegration time test results for enalapril maleate and furosemide tablets were 7.33 ± 3.01 and 11.67 ± 2.25 minutes, respectively. This test result agreed with a study conducted in Turkey [19] and southern Nigerian cities [33] that all brands of enalapril maleate comply with the specification. As in the present study, all brands of furosemide analyzed passed the disintegration test for those studies conducted in Libya [7], Addis Ababa [25] and Bahir Dar, Ethiopia [26].

As used in pharmaceuticals, dissolution is a test that determines the rate of release of a drug from the dosage form into solution forms across the life cycle [35]. In the present study, the dissolution profile showed that almost all brands and batches of enalapril maleate and furosemide met the acceptance criteria. However, one batch from each enalapril maleate and

furosemide brand failed to release as per the pharmacopoeial specification. As in the present study, although two samples failed, almost all brands and batches of enalapril maleate passed the dissolution test for those studies conducted in Brazil [17], Guatemala [18], and Turkey [19]. A study conducted on furosemide tablets in Brazil [20], Argentina [21], Canada [32], Nigeria [23], and Libya [7] also showed results that agreed with the current study. However, a similar study conducted on furosemide drugs in two study areas of Ethiopia, Addis Ababa [25], did not agree with the majority of this study, and Bahir Dar [26] agreed with this major study with respect to the findings of the dissolution test.

The microbial limit test result (TAMC, TYMC, *E. coli*) of furosemide was ＜ 1 CFU/gm (absence of formed colonies), so it passed according to USP <62> specification. This microbial limit test result agreed with a study conducted in Libya in which all the aerobic bacteria and *E. coli* were absent in the five brands of furosemide (< 10 μg/gm of specimen) assessed [7].

Limitations of the Study

The study is performed in certain localized geographic areas; thus, the results might not be applicable to the whole country or in a different context. Nevertheless, the data can be used in situations of similar contexts to fight the worldwide problem of the presence of substandard and falsified (SF) medicines and thereof their public health threat.

Conclusion

The study attempted to evaluate and compare the quality as well as the physicochemical properties of different brands of enalapril maleate and furosemide tablets. Except for the identification and disintegration test, samples from both furosemide and enalapril failed for

pharmacopoeial parameters (assay, friability, dissolution, and uniformity of dosage form) and non-pharmacological parameters (hardness) of the tests performed. For furosemide brands, the microbial limit test in this study revealed that there were no formed colonies of aerobic microbes, yeast, mold and *E. coli*. Generally, the results from this study suggest that approximately half (50.91%) of the enalapril and furosemide products circulating in the markets of the Gedeo and Borena zones at the time of sample collection were substandard (out of specification). Therefore, hypertension medications available on the market may run the risk of losing some of their effectiveness. Hence, it is advisable that the regulatory authority and other stakeholders perform regular actions and monitoring of the medicines available in prone sites such as porous borders to prevent patients from substandard and falsified medicine exposure.

List of Abbreviations and Acronyms

API	Active pharmaceutical ingredients
BP	British Pharmacopoeia
CV	Cardiovascular
CFU	Colony Forming Unit
HPLC	High-pressure liquid chromatography
HNT	Hypertension
mmHg	Millimeter of mercury
NCDs	Noncommunicable diseases
RSD	Relative standard deviation
TAMC	Total aerobic microbial count
TYMC	Total yeast and mold count

USP	United States Pharmacopoeia
UV–Vis	Ultraviolet–visible Spectrophotometer
WHO	World Health Organization

Declarations

Data Sharing Statement

The datasets used in this publication are available upon reasonable request from the corresponding author.

Acknowledgments

The authors would like to thank Dilla University and Addis Ababa University School of Pharmacy, Pharmaceutical Chemistry Department for financial support. We dully thank Humanwell Pharmaceutical Ethiopia PLC for their kind cooperation during medicine quality assessment test studies.

Disclosure

The authors declare that they did not have any conflicts of interest to declare in relation to this study and publication. The funders did not have any role in the study design, data interpretation or publication.

Funding

The study was sponsored by the Addis Ababa University Graduate research support program to ET and the Ministry of Innovation and Technology, government of Ethiopia, by the project scheme National Problem Solving award to AA.

Ethics approval and consent to participate

Ethical approval was obtained from the School of Pharmacy, Addis Ababa University Ethical Review Board (ERB) referenced as ERB/SOP/361/13/2021. Consents and other ethical aspects of the work had been guided on the current principles of research.

Consent for publication

Not applicable

Authors' contributions

All authors made substantial contributions to the conception and design, acquisition of data, or analysis and interpretation of data; ET and AA took the initial steps in conceiving the study. ET, AA, and TE worked in developing the proposal and critical review of the quality assessment tests and sampling site locations; ET carried out sample collection and undertook the quality assessment study. FG assisted in the laboratory work. AA supervised all aspects of the work from sample collection to laboratory testing and mobilized the needed resources and funding for the project. ET: drafted the original manuscript. AA edited and reviewed the manuscript. All the authors have read and approved the final manuscript.

Supplementary file: An additional supplementary file is included as figures and tables to describe details for some methods and results obtained in this study.

References

1. World Health Organization. Global status report on noncommunicable diseases. World Heal Organ. 2010;53(9):1689–99.

2. Mills K, Stefanescu A, He J. The global epidemiology of hypertension. Nat Rev Nephrol. 2020;16(4):223–37.

3. Law MR, Morris JK, Wald NJ. Use of blood pressure lowering drugs in the prevention of cardiovascular disease : meta-analysis of 147 randomised epidemiological studies. Br Med J. 2009;338:1–19.

4. Belayneh B., Moti M., Abreha M. *et al*. Cross-Border Contraband Trade across the Main Route from Moyale to Hawassa: An Exploration into the Causes and Consequences. JBAS. 2015;7(2):10–1.

5. Paul B. and Habtamu E. A Review of Cross-Border Trade in the Horn of Africa. 2021;13–4.

6. Moncloa F, Sromovsky JA, Walker JF, *et al*. Enalapril in Hypertension and Congestive Heart Failure Overall Review of Efficacy and Safety. Drugs. 1985;30(1):82–9.

7. Siaan MM, Altketik KA, Almarzouki A. Evaluation of the Pharmaceutical Quality of Some Furosemide Tablet Brands. Int J Pharm Chem Sci. 2015;4(2):238–46.

8. Verbeeck RK, Kanfer I, Löbenberg R, *et al*. Biowaiver Monographs for Immediate-Release Solid Oral Dosage Forms : Enalapril. J Pharm Sci. 2017;106:1933–43.

9. Katzung BG, Masters SB, Trevor AJ. Basic and Clinical Pharmacology,12 Edition New

YorkMc Graw Hill Medical. 2011. 153–179 p.

10. Carlucci G, Giuseppe E, Mazzeo P. Simultaneous determination of enalapril maleate and hydrochlorothiazide in tablets by derivative UV spectrophotometry and high-performance liquid chromatography. Int J Pharm. 1993;93:245–8.

11. Patchett AA. The chemistry of enalapril. Br J Clin Pharmacol. 2015;18(1984):201–7.

12. WHO. Proposal to waive in vivo bioequivalence requirements for WHO Model List of Essential Medicines immediate-release, solid oral dosage forms. 2006. 1–47 p.

13. Malha L, Mann S. Loop Diuretics in the Treatment of Hypertension. Curr Hypertens Rep. 2016;18(27):1–10.

14. Bader M, Dham R. Bioequivalence evaluation of two brands of furosemide 40mg tablets (Salurin and Lasix) in healthy human volunteers. Biopharm Drug Dispos. 2003;24:245–9.

15. Boles L, Schoenwald R. Furosemide A Pharmacokinetic/ Pharmacodynamic Review (Part I). Clin Pharmacokinet. 1990;18(5):381–408.

16. Kumar M, Bhatt S, Saini V. Comparative Evaluation of Two Different Marketed Brands of Enalapril maleate. J Drug Deliv Ther. 2018;8:265–8.

17. Medeiros C, Ferreira M. Reference, Generic and Smilar Drugs: Quality Assessment of Captopril and Enalapril tablets. Rev Disseminação Científica Sena Aires. 2019;8(1):49–61.

18. Mendoza M, Marroquín MN, Vásquez A, *et al*. In vitro therapeutic equivalence of 20 mg enalapril maleate tablets of national production. Rev Cient. 2020;29(2):2224–5545.

19. Özden S. Quality control studies on enalapril maleate tablets available on the turkish drug market. J Fac Pharm Ankara Univ. 2001;30(1):1–16.

20. Ferreira MS, Viana LC, Matos RA, *et al*. Comparative In Vitro Analysis of Dissolution Profiles of Furosemide Tablets Marketed in Bahia, Brazil. Lat Am J Pharm. 2016;35(9):2064–70.

21. Han YK, Simionato LD, Calvo RG, *et al*. Comparison of Dissolution Profiles Available in the Argentinian Market of Furosemide Tablets. J Appl Solut Chem Model. 2014;3:186–93.

22. Brevedan M, Vidal G. Pharmaceutical equivalence and stability of furosemide tablets in Argentina. Dissolution Technol. 2019;26(4):30–7.

23. Chinaka C, Nwachukwu N. Comparative In Vitro Quality Assessment of Five Brands of Furosemide Tablets Marketed in Port Harcourt, Nigeria. Niger J Pharm Res. 2017;13(2):97–104.

24. Antignac M, Diop BI, Do B, *et al*. Quality assessment of 7 cardiovascular drugs in 10 sub-Saharan countries: The SEVEN study. JAMA Cardiol. 2017;2(2):223–5.

25. Dires T. A comparative in vitro evaluation of anti-hypertensive drugs products (methyldopa, furosemide and propranolol tablets) from local market, Addis Ababa. 2005;1–82.

26. Abebe S, Ketema G, Kassahun H. In vitro comparative quality assessment of different brands of furosemide tablets marketed in northwest Ethiopia. Drug Des Devel Ther. 2020;14:5119–28.

27. United State Pharmacopoeia. Tool for Visual Inspection of Medicines. 2006;1–4.

28. USP. United States Pharmacopeia. USP Monographs, Enalapril Maleate Tablets. USP 43/NF 38. Rockville, MD; 2020.

29. BP. British Pharmacopeia. General Monographs, Furosemide Tablet. 2020.

30. USP. United States Pharmacopeia. USP Monographs, Furosemide Tablets. USP 43/NF 38. Rockville, MD; 2020.

31. USP. United State Pharmacopoeia, Micobiological Examination of Nonsterile Products: Tests for Specified Microorganisms <62>. Rockville, MD; 2020.

32. Qureshi SA, McGilveray IJ. Assessment of Pharmaceutical Quality of Furosemide Tablets from Multinational Markets. Drug Dev Ind Pharm. 2015;24(11):995–1005.

33. Ukwueze SE, Anthony DN. An Ultraviolet-Visible (UV-vis) spectrophotometric determination of pharmaceutical equivalence of some enalapril maleate tablet generics marketed in Nigeria. Pharm Chem J. 2018;5(2):129–35.

34. Natoli D, Levin M, Tsygan L, *et al*. Development, optimization, and scale-up of process parameters: Tablet compression. Elsevier Inc.; 2017. 917–951 p.

35. Hasan M, Rahman M, Islam R, *et al*. A key approach on dissolution of pharmaceutical dosage forms. Pharma Innov J. 2017;6(9):168–80.

Figure
Click here to access/download;Figure;List of Figuresok18.10.2023.doc

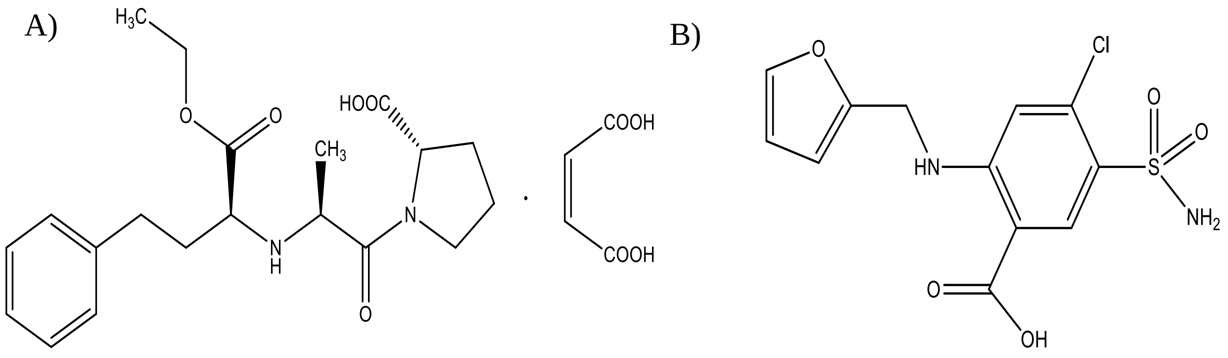

Figure 1.Structural formulae of (A) enalapril maleate and (B) furosemide

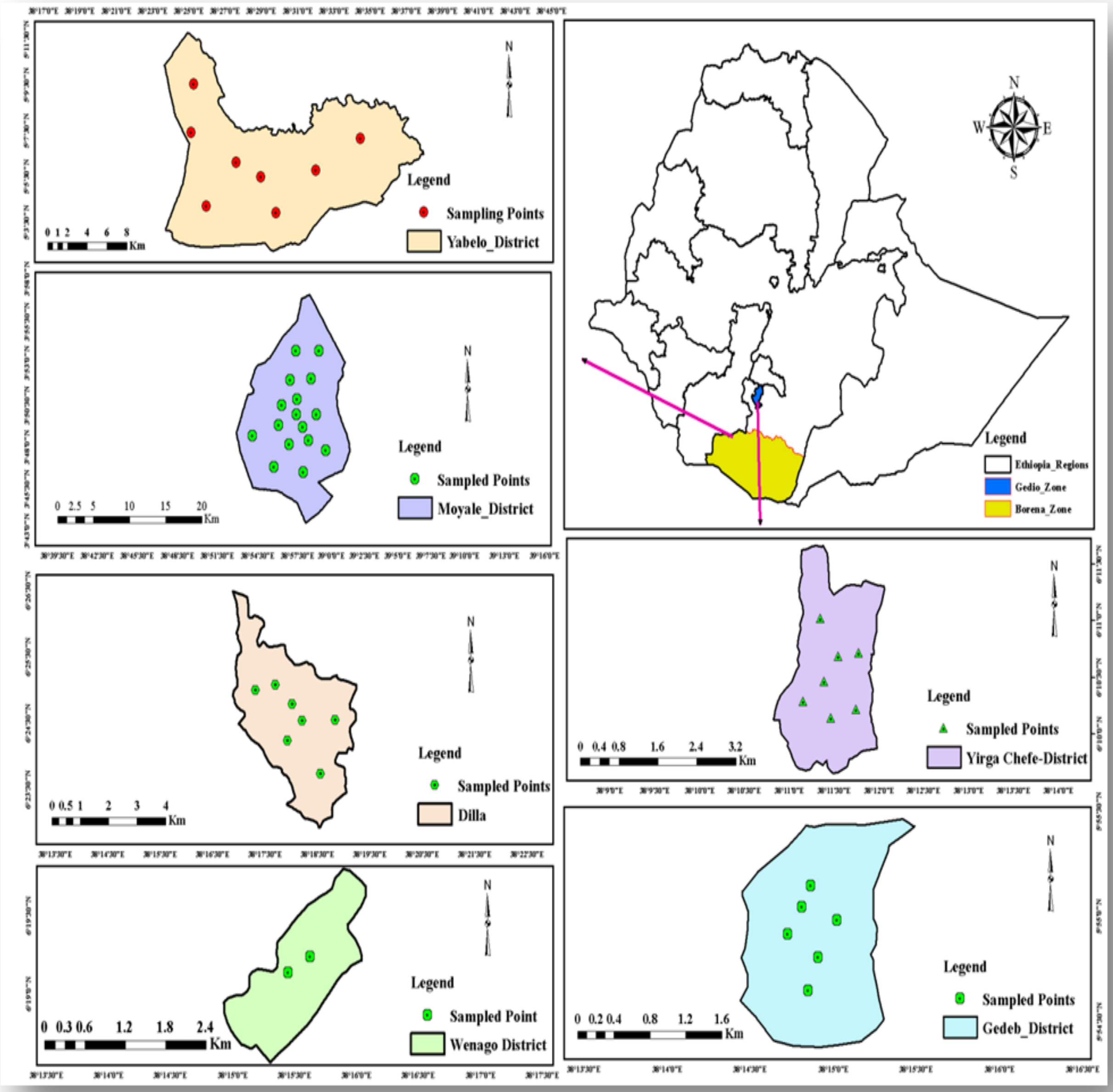

Figure 2. Map of the study area (Gedeo and Borena zones)

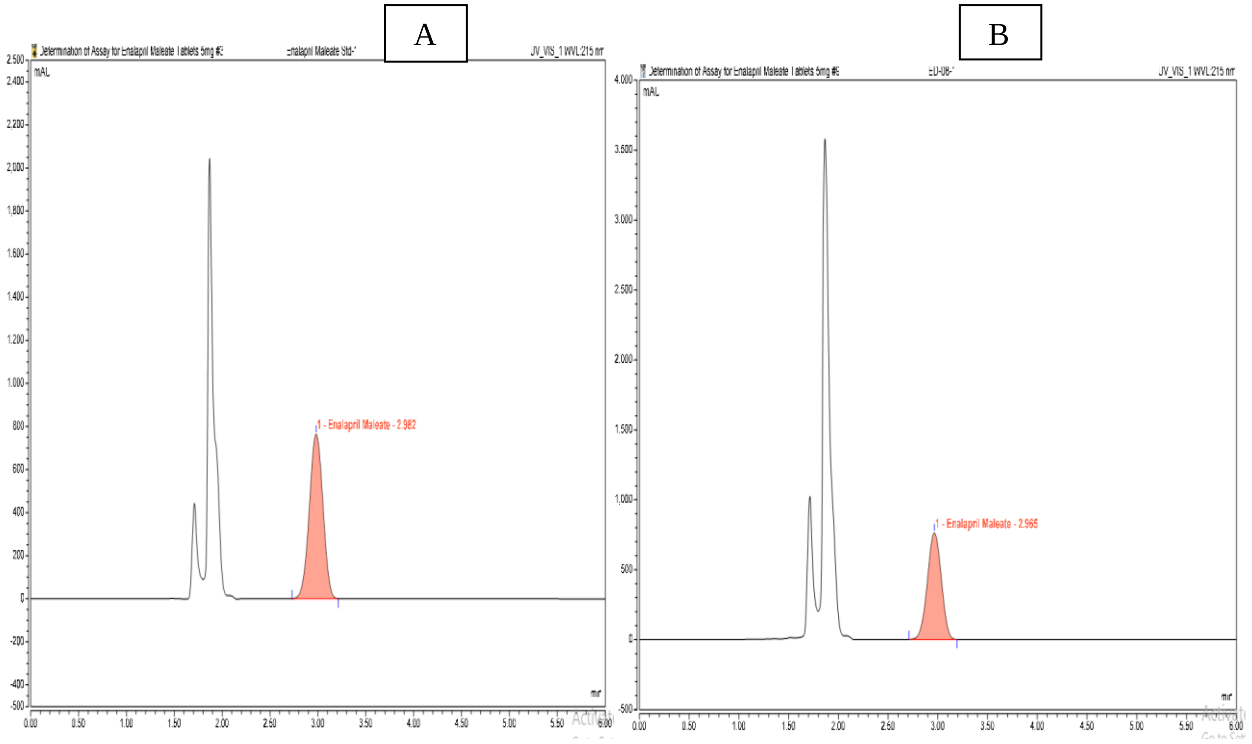

Figure 3. (A) Peak retention time of standard enalapril maleate and (B) sample EM-10

A)

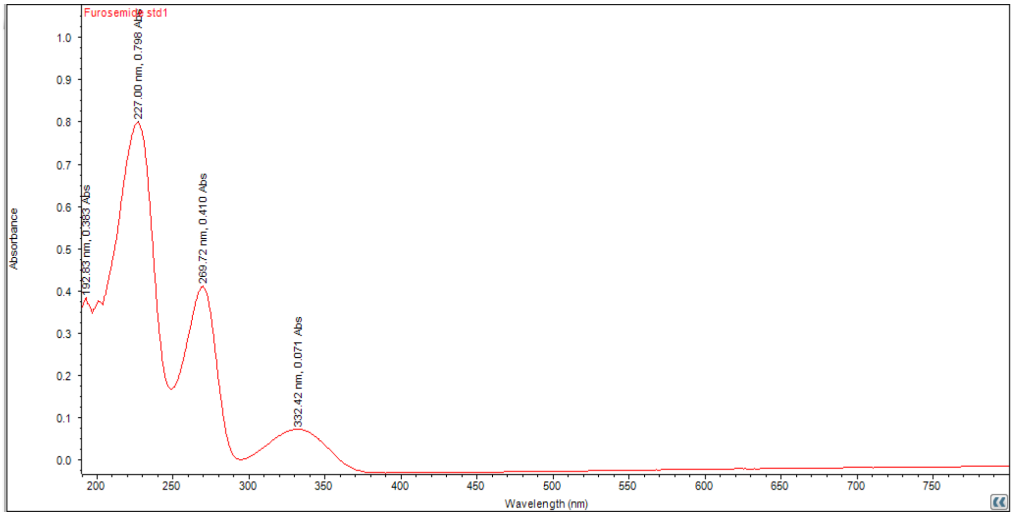

B)

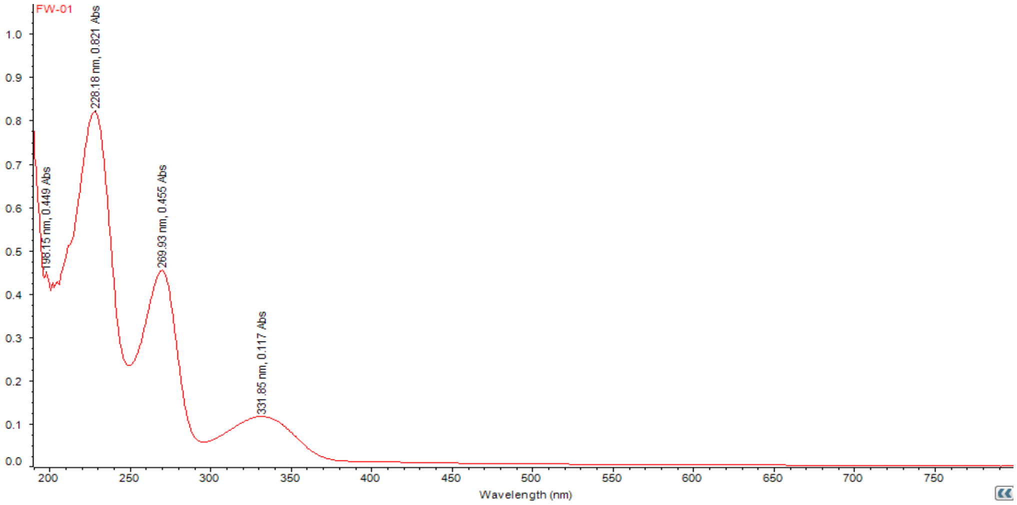

Figure 4. (A) Identification test result of furosemide standard and (B) FW-01 (USP-2020)

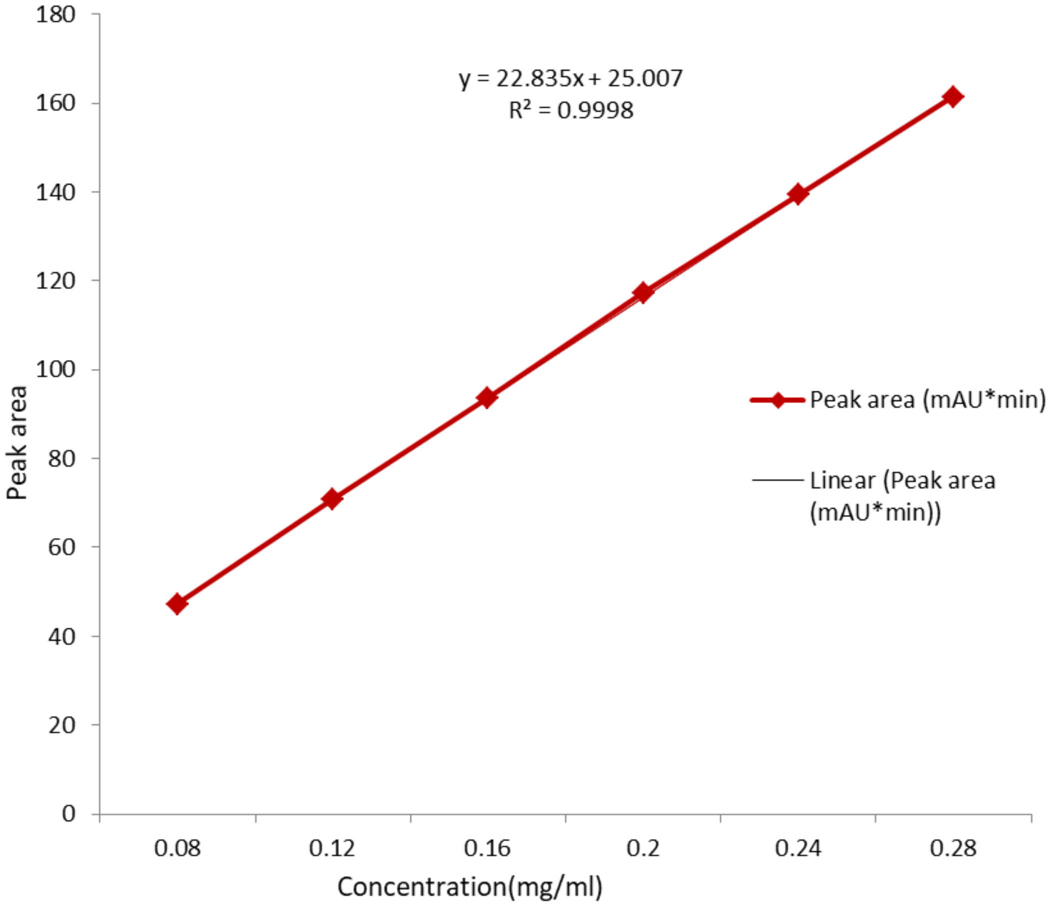

Figure 5. The assay calibration curve of enalapril maleate RS (USP-2020)

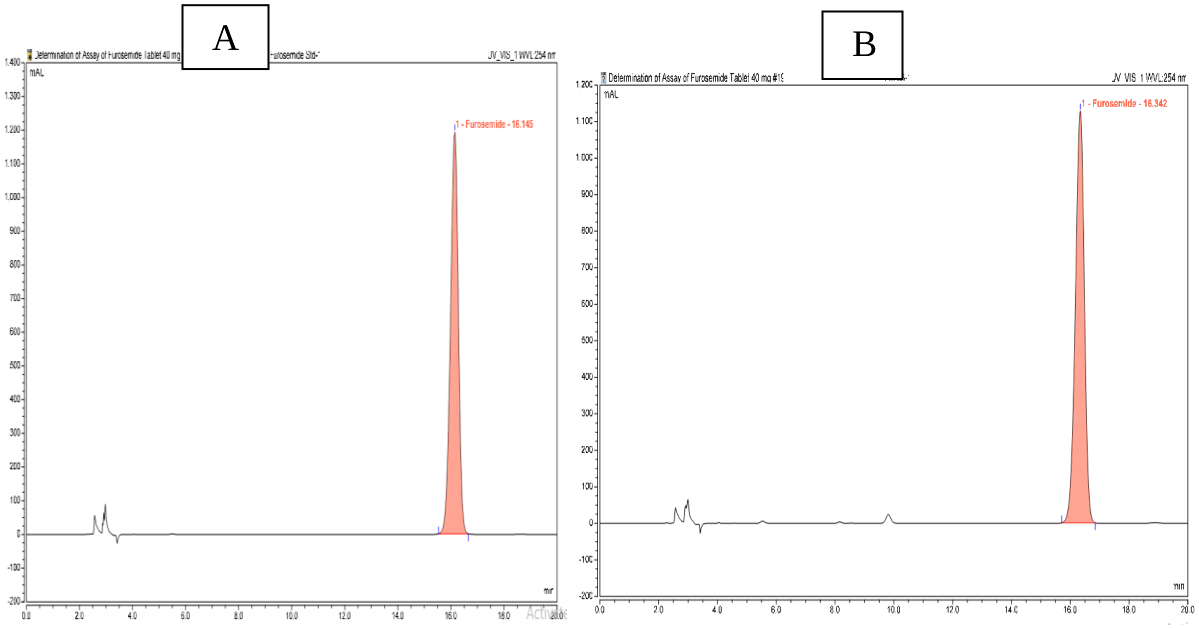

Figure 6. HPLC chromatogram results of furosemide standard (A) and sample FM-06 (B)

A)

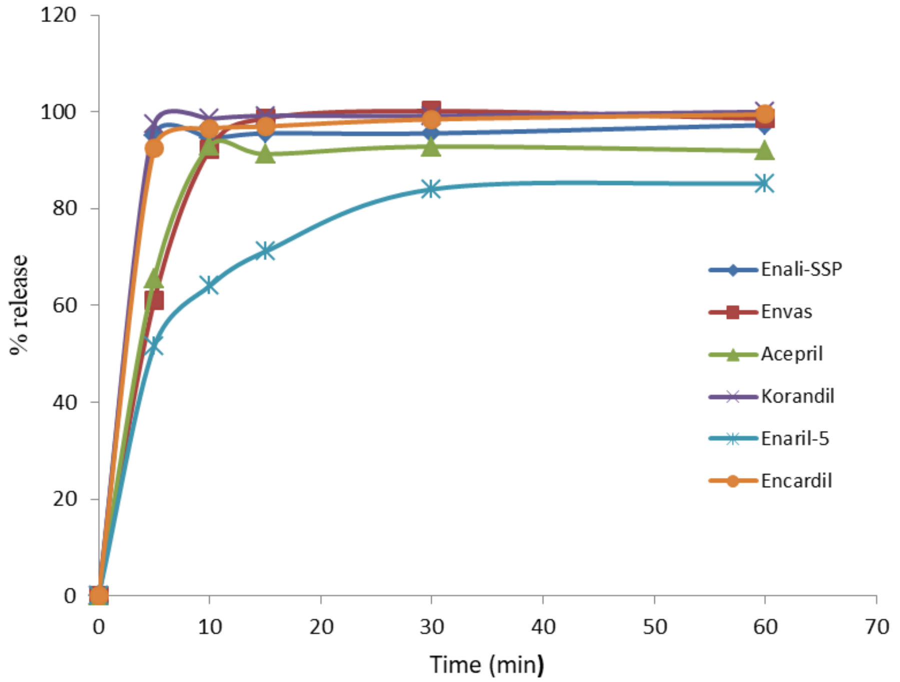

B)

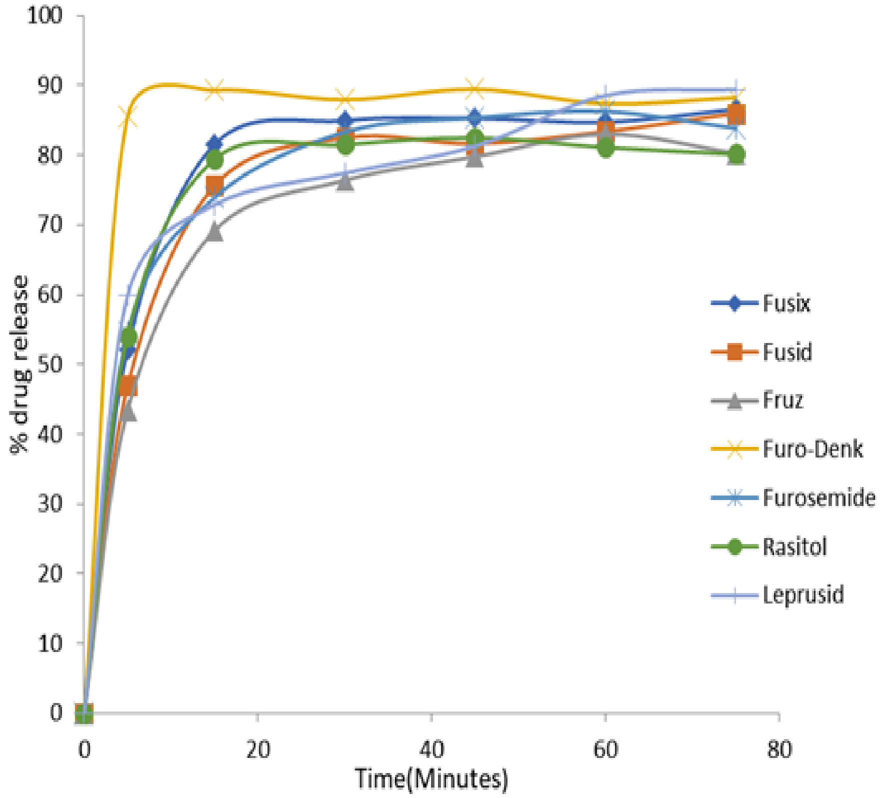

Figure 7. Time dependent dissolution profiles of different brand of (A) enalapril, and (B) furosemide tablets

Click here to access/download
Supporting Information
Supplementary filever2okPLOS global health.doc
