# Supplementary material for: Quality of medicines for Cardio-Vascular Diseases (CVDs) in the Ethiopian border with Kenya: The case of enalapril maleate and furosemide tablet quality in Borena and Gedeo zones
Source: PLOS Glob Public Health. 2024 Jul 15;4(7):e0003104. doi: 10.1371/journal.pgph.0003104 (PMC11249254; doi:10.1371/journal.pgph.0003104)
Supplement: Attachment — Submitted filename: Rebuttal letter.docx [file pgph.0003104.s021.docx]

**March 24, 2024**

**Editor**

**PLOS Global Public Health**

**Dear Editor:**

First and foremost, we want to thank the editor and the reviewers for the very valuable scientific comments on our manuscript that enable d us to improve its scientific merit.

Here are point by point responses to the issues raised

1. Issues raised by the editor:

I: references list:

Please review your reference list to ensure that it is complete and correct. If you have cited papers that have been retracted, please include the rationale for doing so in the manuscript text, or remove these references and replace them with relevant current references. Any changes to the reference list should be mentioned in the rebuttal letter that accompanies your revised manuscript. If you need to cite a retracted article, indicate the article’s retracted status in the References list and also include a citation and full reference for the retraction notice

Answer: The references used are complete and correct to our best. There is no any retracted paper included as reference.

II: figures

Please provide separate figure files in .tif or .eps format only and remove any figures embedded in your manuscript file. Please also ensure all files are under our size limit of 10MB.

For more information about figure files please see our guidelines:

<https://journals.plos.org/globalpublichealth/s/figures> 
<https://journals.plos.org/globalpublichealth/s/figures#loc-file-requirement>

Answer: The figures are now prepared in tiff format in compliance with the journal’s requirements

III. Potential Copyright Issues:

Some material included in your submission may be copyrighted. According to PLOS’s copyright policy, authors who use figures or other material (e.g., graphics, clipart, maps) from another author or copyright holder must demonstrate or obtain permission to publish this material under the Creative Commons Attribution 4.0 International (CC BY 4.0) License used by PLOS journals. Please closely review the details of PLOS’s copyright requirements here: PLOS Licenses and Copyright. If you need to request permissions from a copyright holder, you may use PLOS's Copyright Content Permission form.

Please respond directly to this email or email the journal office and provide any known details concerning your material's license terms and permissions required for reuse, even if you have not yet obtained copyright permissions or are unsure of your material's copyright compatibility.

Potential Copyright Issues:

Fig 2: please (a) provide a direct link to the base layer of the map (i.e., the country or region border shape) and ensure this is also included in the figure legend; and (b) provide a link to the terms of use / license information for the base layer image or shapefile. We cannot publish proprietary or copyrighted maps (e.g. Google Maps, Mapquest) and the terms of use for your map base layer must be compatible with our CC-BY 4.0 license.

Note: if you created the map in a software program like R or ArcGIS, please locate and indicate the source of the basemap shapefile onto which data has been plotted.

If your map was obtained from a copyrighted source please amend the figure so that the base map used is from an openly available source. Alternatively, please provide explicit written permission from the copyright holder granting you the right to publish the material under our CC-BY 4.0 license.

Please note that the following CC BY licenses are compatible with PLOS license: CC BY 4.0, CC BY 2.0 and CC BY 3.0, meanwhile such licenses as CC BY-ND 3.0 and others are not compatible due to additional restrictions.

If you are unsure whether you can use a map or not, please do reach out and we will be able to help you. The following websites are good examples of where you can source open access or public domain maps: 
* U.S. Geological Survey (USGS) - All maps are in the public domain. (<http://www.usgs.gov>) 
* PlaniGlobe - All maps are published under a Creative Commons license so please cite “PlaniGlobe, <http://www.planiglobe.com>, CC BY 2.0” in the image credit after the caption. (<http://www.planiglobe.com/?lang=enl>) 
* Natural Earth - All maps are public domain. (<http://www.naturalearthdata.com/about/terms-of-use/>)

Answer: The figures are made by ourselves and are in compliance with the copy wright rules.

The map in figure 2 is made using the QGIS software ( <https://www.qgis.org/en/site/>). It is in public domain and described as “all are all content is licensed under [Creative Commons Attribution-ShareAlike 3.0 licence (CC BY-SA)](http://creativecommons.org/licenses/by-sa/3.0/)” in their web site. Hence the legal terms allow use of it freely in the publications under 4 cc license too. It is compatible with PLOS CC By 4.0

4. Data availability statement:

In the online submission form, you indicated that "The datasets used in this publication are available upon reasonable request from the corresponding author". All PLOS journals now require all data underlying the findings described in their manuscript to be freely available to other researchers, either 1. In a public repository, 2. Within the manuscript itself, or 3. Uploaded as supplementary information.
This policy applies to all data except where public deposition would breach compliance with the protocol approved by your research ethics board. If your data cannot be made publicly available for ethical or legal reasons (e.g., public availability would compromise patient privacy), please explain your reasons by return email and your exemption request will be escalated to the editor for approval. Your exemption request will be handled independently and will not hold up the peer review process, but will need to be resolved should your manuscript be accepted for publication. One of the Editorial team will then be in touch if there are any issues.

Answer: The following statement had been included in the manuscript as well as in the online submission: **“The datasets used in this publication are included in the manuscript and the supplementary files”.**

**Response to Reviewer 1:**

Reviewer #1: The study conducted addresses the scope and objectives of the study. The results section could have been summarized briefly as the tables and figures are self explanatory. Since the results section is quite elaborate, the discussion section has a lot of redundancy with the results statements.

**Answer: Thank you for the positive comments mentioned above. Regarding redundancy, we had deleted many paragraphs and sentences in the revised manuscript to avoid it. ( eg. Discussion section lineS 461-463)**

**Response to Reviewer 2:**

The manuscript is well written manuscript with some minor issues to be addressed before published.
1. Some of the references cited in this manuscript are dated very old and need to be replaced with the latest ones.

Answer: The references used in some cases might be counted as old as it deals with principles nevertheless our references are not as such old. We used only 4 references in the 1990’s otherwise all our references used are in 2000’s. As we deal with facts and widely established science medicine quality, it is proper to use some references in the 1990’s in themes related to established facts and methodologies.
2. Its been mentioned in the manuscript that the enalapril and the furosemide are the widely prescribed medicines for hypertension. No guidelines for hypertension made such a recommendation. The information provided in the manuscript need to be correct and accurate. Furosemide is a loop diuretic commonly used to manage conditions like edema and congestive heart failure. While it can lower blood pressure, it is not a first-line antihypertensive agent.
Loop diuretics, including furosemide, are not recommended as the primary treatment for hypertension due to the lack of robust outcome data.

Answer: Guidelines and medical practices sometimes slightly vary from country to country but considering the comment our manuscript had revised taking this comment keenly. (please have a look at line 24 ff). our intention previously was to refer their wide use but not as first line treatment options but as the sentences can be understood in such sense it is modified in the revision “…… as among the drugs prescribed”…….
3. The grammatical mistakes found throughout the manuscript decreases the weightage of the manuscript.

Answer: In our revisions we had worked on the language issue exhaustively and such issues are addressed (please have a look at lines 32, 537 etc…)

Sincerely,

Ayenew Ashenef

On Behalf of all authors
